# Genomewide association study in cervical dystonia demonstrates possible association with sodium leak channel

**DOI:** 10.1002/mds.25732

**Published:** 2013-11-13

**Authors:** Kin Y Mok, Susanne A Schneider, Daniah Trabzuni, Maria Stamelou, Mark Edwards, Dalia Kasperaviciute, Stuart Pickering-Brown, Monty Silverdale, John Hardy, Kailash P Bhatia

**Affiliations:** 1 Department of Molecular Neuroscience, University College London (UCL) Institute of NeurologyLondon, United Kingdom; 2 Department of Neurology, University of KielKiel, Germany; 3 Sobell Department of Motor Neuroscience and Movement Disorders, University College London (UCL) Institute of NeurologyLondon, United Kingdom; 4 Department of Clinical and Experimental Epilepsy, University College London (UCL) Institute of NeurologyLondon, United Kingdom; 5 University of Manchester, Institute of Brain, Behaviour and Mental HealthManchester, United Kingdom; 6 Greater Manchester Neuroscience Centre, Salford Royal Foundation TrustGreater Manchester, United Kingdom; 7 Reta Lila Weston Research Laboratories, University College London (UCL) Institute of NeurologyLondon, United Kingdom

**Keywords:** cervical dystonia, GWAS, imputation, sodium leaking channel, *NALCN*

## Abstract

Dystonia is a common movement disorder. A number of monogenic causes have been identified. However, the majority of dystonia cases are not explained by single gene defects. Cervical dystonia is one of the commonest forms without genetic causes identified. This pilot study aimed to identify large effect-size risk loci in cervical dystonia. A genomewide association study (GWAS) was performed. British resident cervical dystonia patients of European descent were genotyped using the Illumina-610-Quad. Comparison was made with controls of European descent from the Wellcome Trust Case Control Consortium using logistic regression algorithm from PLINK. SNPs not genotyped by the array were imputed with 1000 Genomes Project data using the MaCH algorithm and minimac. Postimputation analysis was done with the mach2dat algorithm using a logistic regression model. After quality control measures, 212 cases were compared with 5173 controls. No single SNP passed the genomewide significant level of 5 × 10^−8^ in the analysis of genotyped SNP in PLINK. Postimputation, there were 5 clusters of SNPs that had *P* value <5 × 10^−6^, and the best cluster of SNPs was found near exon 1 of *NALCN*, (sodium leak channel) with *P* = 9.76 × 10^−7^. Several potential regions were found in the GWAS and imputation analysis. The lowest *P* value was found in *NALCN*. Dysfunction of this ion channel is a plausible cause for dystonia. Further replication in another cohort is needed to confirm this finding. We make this data publicly available to encourage further analyses of this disorder.

Dystonia is a “syndrome of sustained muscle contractions, frequently causing twisting and repetitive movements or abnormal postures.”^1^ It is a common movement disorder with an estimated prevalence of 430 per million in the Northern England survey.^2^,^3^ The Epidemiological Study of Dystonia in Europe (ESDE) Collaborative Group showed crude annual prevalence rate of 152 per million across 8 European countries.^4^ The real frequency may be higher as this study might have underestimated the prevalence secondary to underascertainment of cases. An American study online survey on cervical dystonia suggested a prevalence of up to 0.28%.^5^

Clinical presentation of dystonia is heterogeneous, from focal involvement such as cervical dystonia to generalized torsional dystonia. At present, there are over 20 dystonia loci named, *DYT1* to *DYT25*. Among these, *DYT1*, *DYT3*, *DYT5*, *DYT6*, *DYT8*, *DYT10*, *DYT11*, *DYT12*, *DYT16*, *DYT18*, *CIZ1* (*DYT23*), *ANO3* (*DYT24*), and *GNAL* (*DYT25*) have been cloned.^6^–^10^

Cervical dystonia is the most common form of dystonia.^3^,^5^,^11^ A recent Dutch study on focal primary torsion dystonia reported a positive family history in 25% but only 2.4% had Mendelian inheritance pattern among their primary torsion dystonia cases.^11^ Sixty-four percent (64%) of focal dystonia cases in this Dutch study were cervical dystonia. The majority of cervical dystonia are transmitted in non-Mendelian pattern, which suggests cervical dystonia is likely a complex disease rather than monogenic form. Hitherto, there has been no report of a high-density genomewide association study (GWAS) to identify the loci associated with non-Mendelian dystonia. This study was a pilot attempt to identify any large effect-size loci.

## Patients and Methods

Focal cervical dystonia cases were recruited from 2 movement disorders clinics in UK: (1) National Hospital for Neurology and Neurosurgery, London; and (2) Salford Royal Foundation Trust, Manchester. Only cases that remained focal involvement were recruited and most of them were followed up in Botulinum Toxin Clinic. Cases were reviewed by movement disorder specialists, and only presumptive primary cases were recruited, with secondary causes like Wilson’s disease or other neurodegenerative disease ruled out where clinically appropriate. Cases suggestive of Mendelian inheritance were excluded. *DYT1* was excluded where necessary. Because of small sample size, subclassification according to family history of movement disorders and age of onset was not attempted. Only cases of assumed European descent were recruited. All patients gave informed consent to the study, approved by the respective local ethics committee. DNA was extracted locally from blood and genotyped in the University College London Genomics Microarray Centre, using Illumina Human 610-Quad BeadChip (Illumina, San Diego, CA, USA).

Control data of European descent was drawn from the Wellcome Trust Case Control Consortium (WTCCC) data set as previously reported.^12^ A total of 2930 samples from 1958 Birth Cohort and 2737 samples from National Blood Services were genotyped in Illumina 1.2 M Duo array by the Wellcome Trust Sanger Institute. Quality control measure was performed both before and after merging with the cases as detailed in the Supporting Materials.

Genotyped data from cervical dystonia cases was assembled in GenomeStudio (v2011.1) per the manufacturer’s suggestion (Illumina). Postassembly quality control was performed in GenomeStudio and PLINK (version 1.07).^13^ In brief, genotyping quality of the sample and single-nucleotide polymorphisms (SNPs) were controlled for in GenomeStudio (Illumina). Further checking for gender mismatch, sample relatedness (identity-by-descent, excluding piHat >0.125) (Pi-Hat — a parameter in PLINK using estimates of pairwise IBD to find pairs of individuals who are possibly related.), Hardy-Weinberg equilibrium (excluding *P* < 1× 10^−4^), allele frequency (excluding minor allele frequency [MAF] <0.01), nonrandom missingness (missingness-by-haplotype, excluding *P* <1 × 10^−4^; missingness-by-genotype, excluding *P* <1 × 10^−4^) and population substructure (excluding 6 SD from combined mean of Northern and Western European ancestry (CEU) and Toscani in Italia (TSI) in multidimensional scaling [MDS] component 1 or 2) were performed in PLINK^13^ (details in Supporting Materials).

The dystonia sample data was merged with the Wellcome Trust Case Control Consortium (WTCCC), and association analysis was performed using logistic regression in the PLINK package.^13^ Three covariates were used in the regression: gender and the first 2 components of PLINK MDS analysis to adjust for gender and genetic variation. Because of our relatively small sample size, we did not perform separate analysis that looked specifically at known loci.

Imputation for autosomal chromosome was performed with MaCH^14^ and minimac,^15^ in chunks of 10 megabases with 1 megabase overlapping at both ends. Other parameters were as suggested by authors (details in the Supporting Materials). Postimputation association was done in mach2dat^16^ excluding imputed SNPs with *r* squared (RSQR) <0.3 as suggested. SNPs with MAF <0.03 were not entered into the final analysis. A higher cutoff was chosen as the small sample size in this study contributed to false-positive association in SNPs with low MAF.^17^ Reference for imputation was taken from the 1000 Genomes Project 2010 August release and composed of 283 individuals from the European continental group.^18^

Further statistical analysis and plotting were done in R (version 2.11.1).^19^ Plots of the identified regions were made with Locus Zoom.^20^

## Results

**Figure 1:**
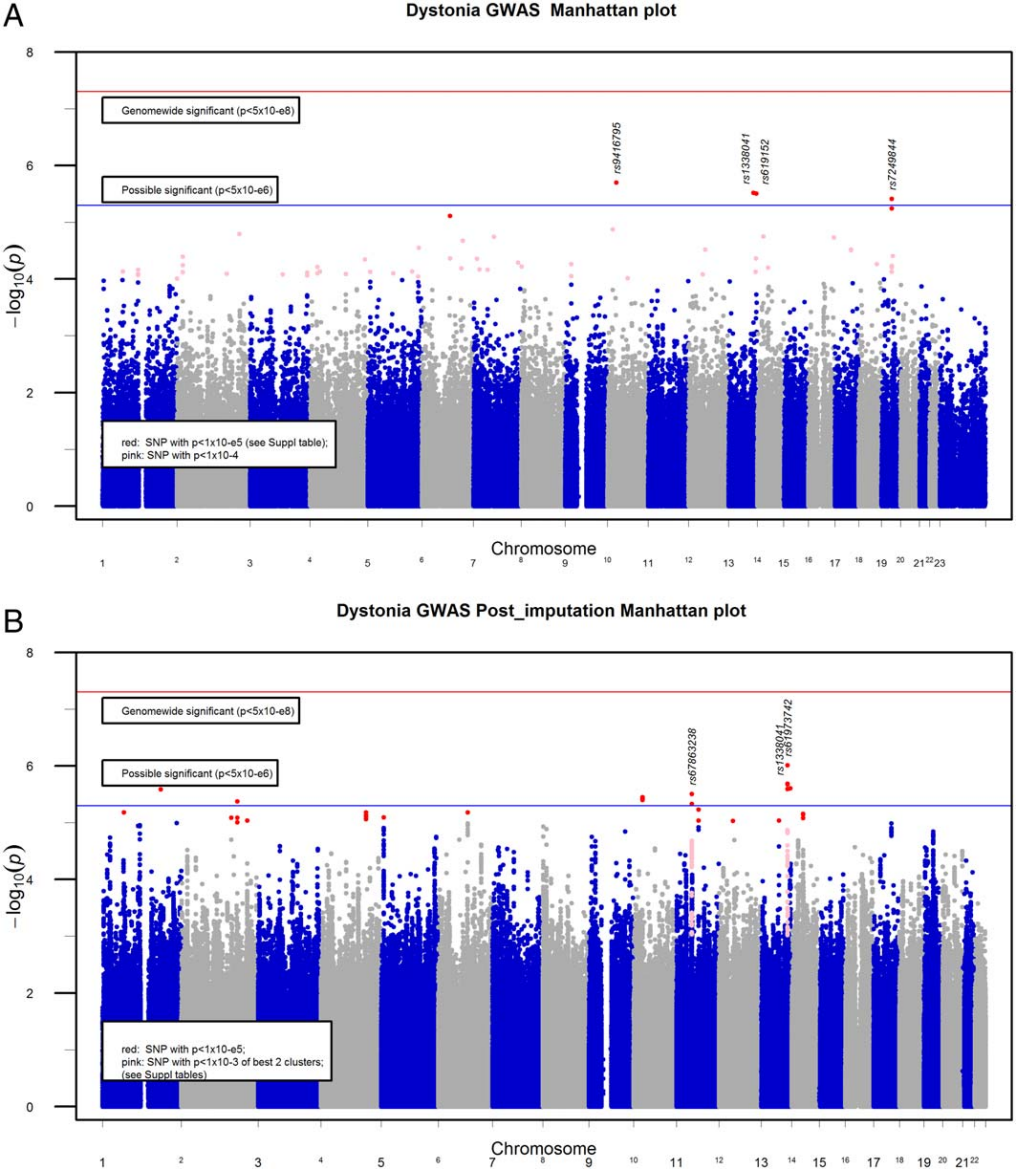
(A) Manhattan plot of GWAS. (B) Manhattan plot of imputed result. The SNPs with *P* < 0.001 in the best 2 regions were labeled in pink, showing clustering of SNPs around the regions. GWAS, genomewide association study; SNP, single-nucleotide polymorphism. [Color figure can be viewed in the online issue, which is available at wileyonlinelibrary.com.]

A total of 233 cases were genotyped and 212 cases (66 M, 146 F, mean age 60.6 years, SD 10.9 years) remained after quality control and 494 k SNPs had MAF >0.01 (exclusion breakdown in Supporting Tables 1 and 2). The cases were clustered around the CEU-TSI controls in the MDS plot confirming European descent (Supporting Fig. 1a-c). These were compared with 5173 controls (2609 M, 2564 F) from WTCCC. Quantile-quantile (QQ) plot did not deviate from the expected (Supporting Fig. 3) and lambda was 1.02. No single SNP had reached a genomewide significant association (defined as *P* < 5 × 10^−8^). The best signal was rs9416795 (*P* = 2.00 × 10^−6^), located in an intergenic region on chromosome 10. This was followed by rs1338041 on chromosome 13, intron region of *NALCN*, coding for sodium leak channel, nonselective (Fig. 1A, Supporting Table 3).

After imputation and quality controls (RSQR >0.3 and MAF ≥0.03), there were 5.67 million SNPs available in chromosomes 1 to 22. These had satisfactory genomewide coverage, except a few regions, mainly telomeric and centromeric regions (Manhattan plot, Fig. 1B). The regions not well covered with GWAS and imputation are listed in Supporting Table 7. There were no SNPs with genomewide significance (defined as *P* < 5 × 10^−8^). A few clusters of possible associations (defined as *P* < 5 × 10^−6^) were found and shown in the Manhattan plot (Fig. 1B, Table 1).

**Table 1:** Postimputation SNPs with *P* value <5 × 10^−6^

SNP	CHR	Position	Major allele	Frequency of major allele	RSQR^a^	Effect 1	OR	SEM	LR chi square	*P* value of LR	Symbol	Predicted function	Splice distance
rs61973742	13	102083273	A	0.9384	0.8881	1.579	4.848	0.415	23.9755	9.76E-07	*NALCN*	5′ Upstream	
rs1338051	13	102062341	G	0.6595	0.9932	−0.481	0.618	0.1	22.5497	2.05E-06	*NALCN*	Intronic	6323
rs9518385	13	102060280	A	0.6597	0.9947	−0.48	0.618	0.1	22.5411	2.06E-06	*NALCN*	Intronic	8384
rs9518384	13	102059871	C	0.6597	0.9951	−0.48	0.619	0.1	22.5246	2.08E-06	*NALCN*	Intronic	8355
rs1338041^b^	13	102058862	A	0.6597	0.9955	−0.48	0.619	0.1	22.4931	2.11E-06	*NALCN*	Intronic	7346
rs619152^b^	13	110939497	G	0.6437	0.9328	−0.489	0.614	0.103	22.1724	2.49E-06	*COL4A1*	Intronic	19794
rs3916908	13	102058054	A	0.6602	0.9949	−0.477	0.621	0.1	22.1374	2.54E-06	*NALCN*	Intronic	6538
rs12132318	1	183797688	T	0.9376	0.3209	−1.422	0.241	0.28	22.0992	2.59E-06	*RGL1*	Intronic	19012
rs67863238	11	48267856	G	0.9439	0.8808	1.608	4.994	0.442	21.7354	3.13E-06	*OR4X2*	3′ Downstream	
rs1249277	10	28720076	G	0.8497	0.9894	−0.574	0.563	0.118	21.5017	3.54E-06		Intergenic	
rs1249281	10	28716177	G	0.8505	0.9956	−0.572	0.564	0.118	21.3706	3.79E-06		Intergenic	
rs9416795^b^	10	28709550	G	0.8508	0.9998	−0.57	0.565	0.118	21.2877	3.95E-06		Intergenic	
rs10930717	2	176742322	G	0.9524	0.9092	−0.896	0.408	0.177	21.1596	4.23E-06	*KIAA1715*	3′ Upstream	
rs35875350	11	48230490	G	0.9433	0.9333	1.513	4.538	0.421	20.9828	4.63E-06	*OR4B1*	5′ Upstream	

a Quality measure of imputation, from 0 to 1, with values <0.3 suggestive of poorly imputed.

b SNP also found in the nonimputed genotype platform.

SNP, single-nucleotide polymorphism; CHR, chromosome; RSQR, *r* squared (*r*^2^); OR, odds ratio; SEM, standard error of the mean; LR, likelihood ratio.

**Figure 2:**
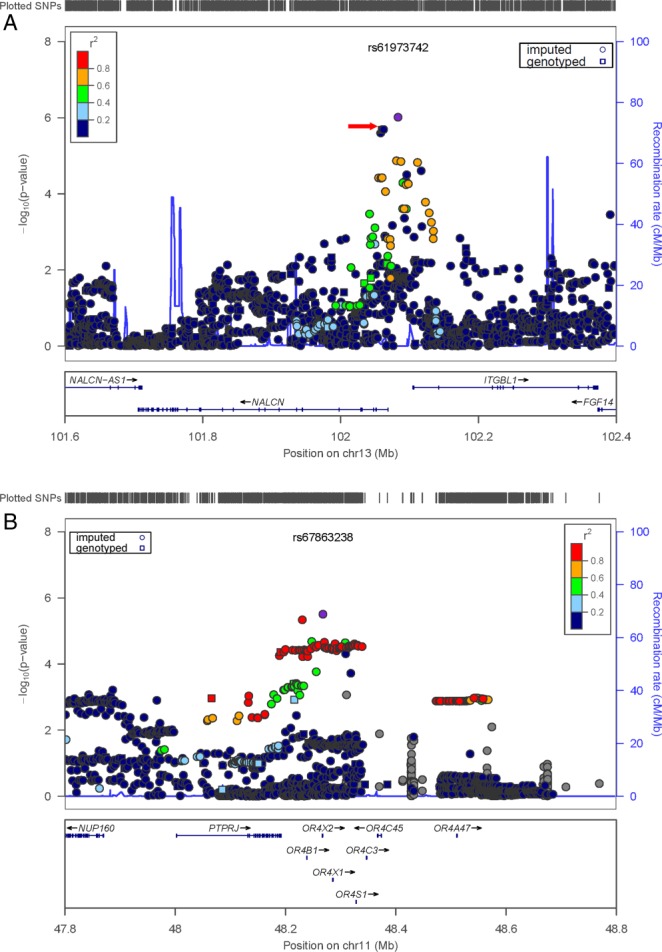
(A) Local association plot with LD of *NALCN* region (Locus Zoom). There is a cluster of imputed SNPs with *P* value suggesting possible association. Red arrow is the best GWAS hit (rs1338041) in this region. (B) Local association plot with LD at Chr 11. Tightly linked imputed SNPs suggest possible association of cervical dystonia with an uncommon haplotype in the region that was not well tagged by common SNPs in GWAS. LD, linkage disequilibrium; GWAS, genomewide association study; Chr, chromosome; SNP, single-nucleotide polymorphism. [Color figure can be viewed in the online issue, which is available at wileyonlinelibrary.com.]

With imputation, the best signals found clustered around *NALCN* with best *P* value of 9.8 × 10^−7^ in rs61973742 and 5 more SNPs in the same gene just short of the best *P* value. NALCN protein is a sodium leaking channel. The majority of the associated SNPs were found in the first intron between the first and second exon. The remaining were within a few kilobases of the 5′ region of exon 1 and 5′ untranslated region (UTR) (Fig. 2A, Supporting Table 6). The second cluster with peak *P* at 3.1 × 10^−6^ was found in rs67863238, chromosome 11 base position 48,267,856 (hg19). This cluster codes for a number of olfactory receptors (*OR4X1*, *OR4X2*, *OR4S1*, and *OR4B1*) (Fig. 2B, Supporting Table 5).

Remaining imputed SNPs that passed the possible associations were found on chromosome (Chr) 1 *RGL1*, Chr 2 intergenic 3′ of *KIAA1715*, Chr 10 intergenic, and Chr 11 *COL4A1*. The SNPs and local plots of these regions are shown in Supporting Table 4 and Supporting Figures 4 and 5.

## Discussion

In this cervical dystonia GWAS, no loci reached a statistically significant association with dystonia. No common SNPs or loci with large effect size were found in dystonia. The most crucial limitation was the small sample size, which was underpowered to detect loci with a smaller disease effect. Assuming MAF of 0.4, odds ratio 1.3, additive model, and prevalence of 430 per million in dystonia, it would need more than 1800 cases to achieve an 80% power.^21^ If the odds ratio was as high as with the Complement Factor H in macular degeneration,^22^ the cohort of 212 cases with same assumptions would have a power of almost 100% to detect the SNP. Hence, we conclude that a common SNP with large effect-size is unlikely to be present in idiopathic cervical dystonia, at least in Chr 1 to 22 as shown in Manhattan plot (Fig. 1) and within the regions that were usually genotyped by microarray (Supporting Table 7 for uncovered regions).

The imputation results showed a few clusters of potential significance. The top hit found in *NALCN* region is interesting. Dysfunction of a sodium channel is a biologically feasible candidate in dystonia. Allelic mutation of *SCN8A* in mice can present with dystonia.^23^ Our group has also recently identified mutations in anoctamin (*ANO3*-*DYT23*), a calcium-gated chloride channel gene, leading to autosomal dominant craniocervical dystonia.^9^ This strongly supports an ion channel as a plausible candidate gene for dystonia. Such channels are also potentially pharmacologically modifiable.

NALCN protein is a member of 4 × 6 transmembrane voltage-independent, nonselective, noninactivating ion channel found in all animals studied and universally expressed in mouse brain and spinal cord.^24^ Our UK Brain Expression Consortium data shows *NALCN* is universal expressed in brain^25^ (Supporting Fig. 3). In schizophrenia, the associated SNP in *NALCN* was rs2044117, located at the last intron.^26^ The C-terminal end of the protein is the important site in coupling with UNC79 and UNC80 proteins.^27^ This complex senses calcium level and results in alteration of leaking current and neuronal excitability, comparable to *ANO3* as a calcium-gated chloride channel. Mice with exon 1 knocked out die from disrupted respiratory neuronal firing in the brainstem,^24^ suggesting regions other than the C-terminal are also critical. Our SNPs were clustered around the exon 1 both at 5′ and within the first intron. They may not necessarily be the pathogenic variants but just tagged SNPs. There are a few synonymous and nonsynonymous SNPs found in the exon 1 (rs144447052, rs145910377, rs74707055, rs76774740, rs75606652, rs77203309, rs188237867, rs79047578, and rs9557636). These SNPs are very rare, with MAF well below 0.01 and not included in the imputation and analysis. These may be pathogenic but unidentified rare pathogenic variants tagged to our SNPs are also possible.

Local plot of postimputation SNPs (Fig. 2A, Supporting Table 6) showed multiple SNPs with possible *P* value identified. This gives support for possible association of this region with cervical dystonia. The best SNP from imputation (rs61973742) has a low *r*^2^ with the best SNP rs1338041 in GWAS. There is a great discrepancy of the MAF, 0.062 in rs61973742 and 0.34 in rs1338041. A discrepancy in MAF can lead to low *r*^2^ (VanLiere and Rosenberg^28^ and Wray^29^). These 2 SNPs have a |D′| = 0.88 despite its low *r*^2^ (0.05) in 1000 Genomes 378 European population. Alternatively, there may be a few haplotypes in the region that are pathogenic. GWAS common SNPs tag to 1 haplotype and others discovered through imputation.

The next potential cluster was in Chr 11. It was not found in the initial GWAS, but multiple SNPs with borderline *P* value were identified from imputation (Supporting Table 5). If replicated, this represents the power of finding new associations that are not well tagged by common SNPs and are identified through haplotypes inferred during imputation. This group of associations, namely *OR4X1*, *OR4X2*, *OR4S1*, and *OR4B1*, is interesting (Fig. 2B). They belong to the olfactory receptor, family 4, a type of G-protein-coupled receptors (GPCRs). Olfactory function may seem unrelated to dystonia. Recently Fuchs et al.^10^ reported the association of *GNAL* mutation with primary torsion dystonia, and the predominant clinical feature in these patients is cervical dystonia. *GNAL*, coding an olfactory G protein [G(olf)] is found highly expressed in striatum and coupled with the expression of dopamine D1 receptor (DRD1).^30^ It is located at chromosome 18p. 18p-deletion was reported to be associated with *DYT7*.^31^,^32^ This association was recently challenged.^33^ The 4 olfactory genes (*OR4X1*, *OR4X2*, *OR4S1*, and *OR4B1*) are universally expressed in brain and *ORB1* is highly expressed in striatum as well (Allen Brain Atlas^34^).

The GWAS and imputation association analysis employed 2 different algorithms. This may lead to slightly different *P* values in GWAS and imputation for the same SNP. The 3 other hits found in GWAS were located at intergenic at Chr 6 92 Mb, Chr 10 28 Mb, and Chr 19 29 Mb. The local plots of postimputation SNPs in these regions are shown in Supporting Figure 4. The sparse imputed SNPs around Chr 10 suggested that might be a false-positive result, as were the imputation finding at Chr 1 183 Mb and Chr 2 176 Mb (Supporting Figure 4). The extra SNPs found with imputation at Chr 6 92 Mb and Chr 19 29 Mb suggested potential association. The *P* value was lower than the best 2 clusters in *NALCN* and *OR4X1* and the functional role within an intergenic region is difficult to predict. Given the recent findings from ENCODE, intergenic region may still play a significant function in transcription.^35^

In summary, we found a plausible association, though not statistically confirmed, of cervical dystonia with SNPs in the *NALCN* region. Replication on another cohort of cervical dystonia cases would be essential to confirm the association. As dystonia GWAS is relatively understudied, we make all these data publicly available to encourage further analyses of the problem.
