# Supplementary figures and images for: Genomewide association study in cervical dystonia demonstrates possible association with sodium leak channel
Source: Mov Disord. 2013 Nov 13;29(2):245–51. doi: 10.1002/mds.25732 (PMC4208301; doi:10.1002/mds.25732)

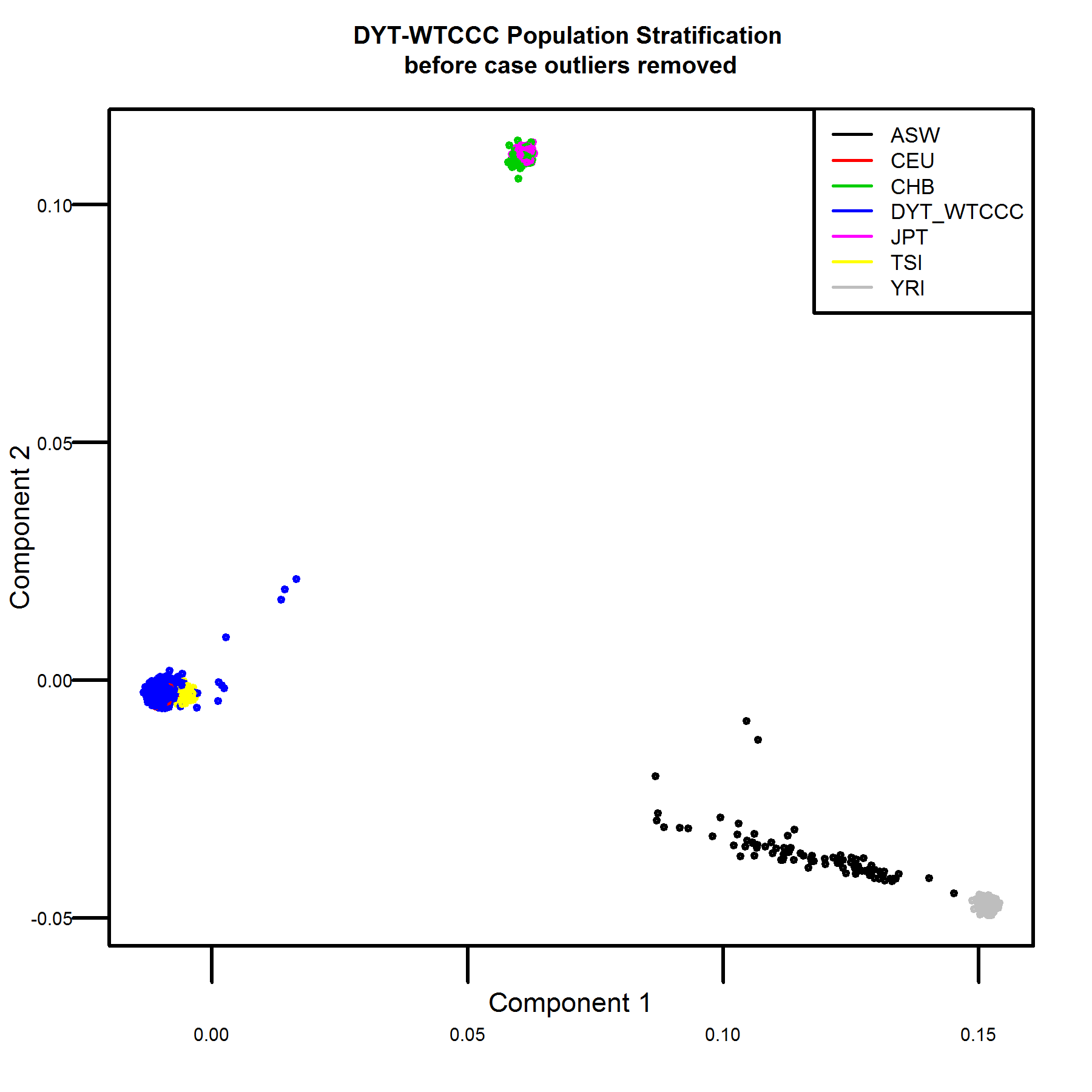

Supplement: Supplementary file 1 [file mds0029-0245-sd1.tif]

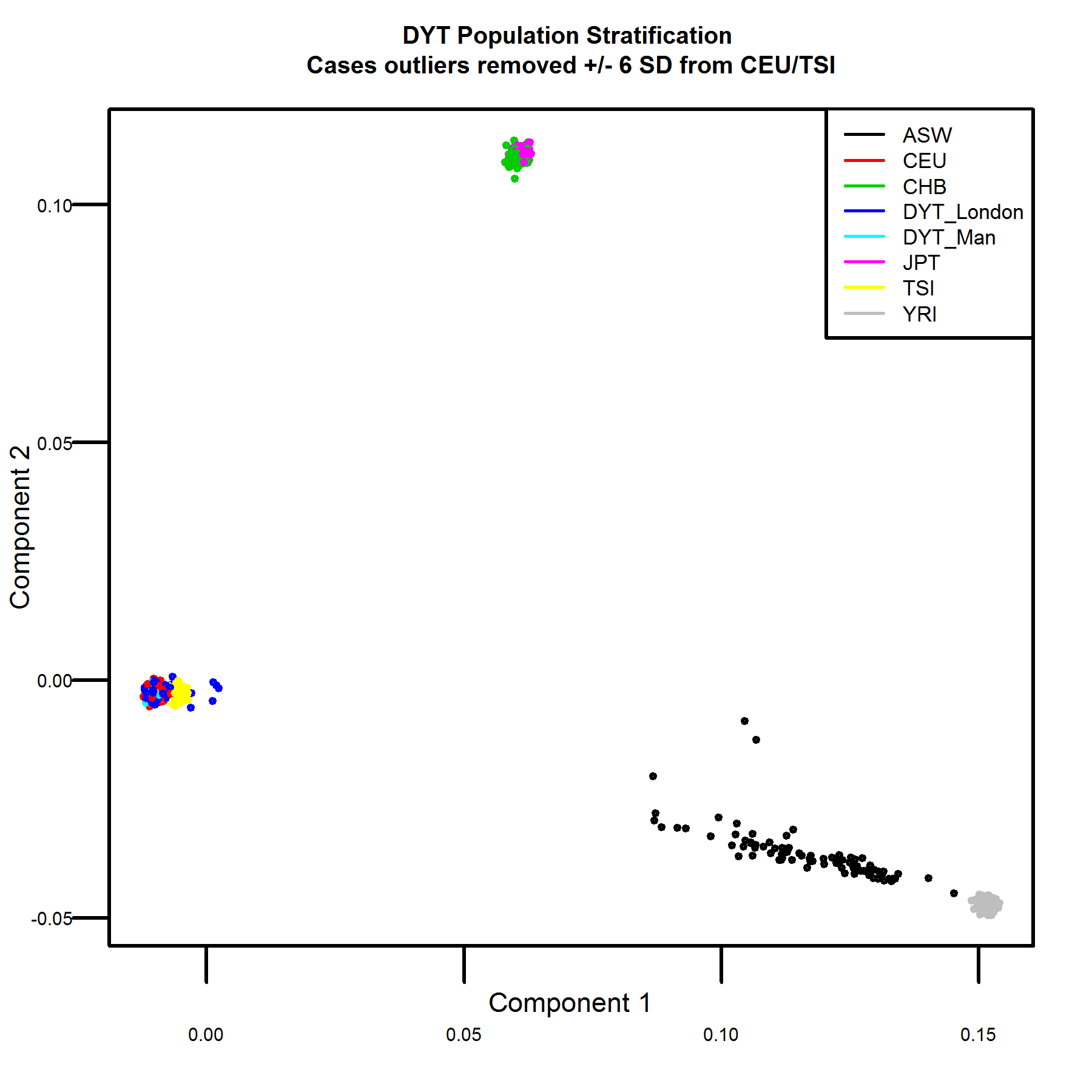

Supplement: Supplementary file 2 [file mds0029-0245-sd2.tif]

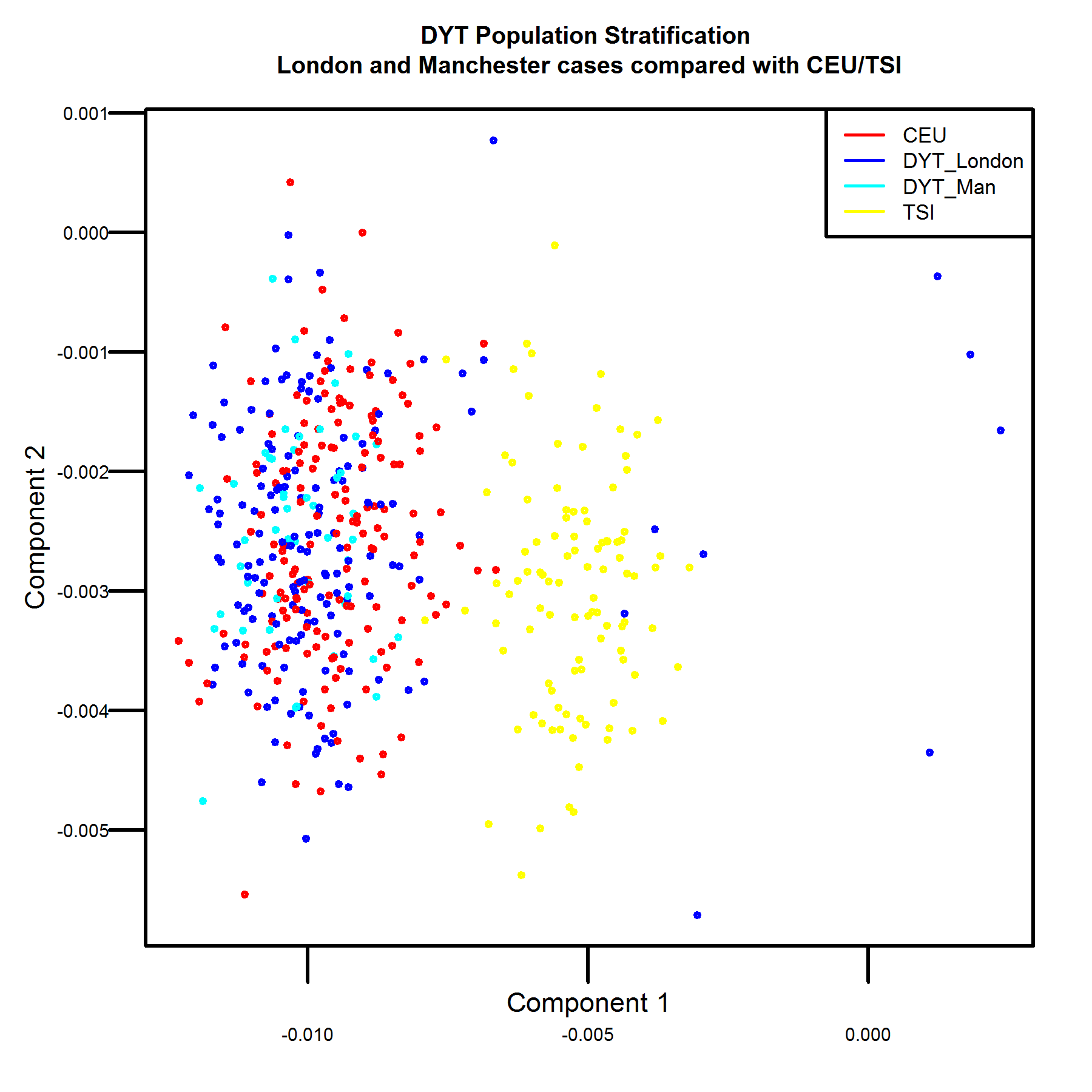

Supplement: Supplementary file 3 [file mds0029-0245-sd3.tif]

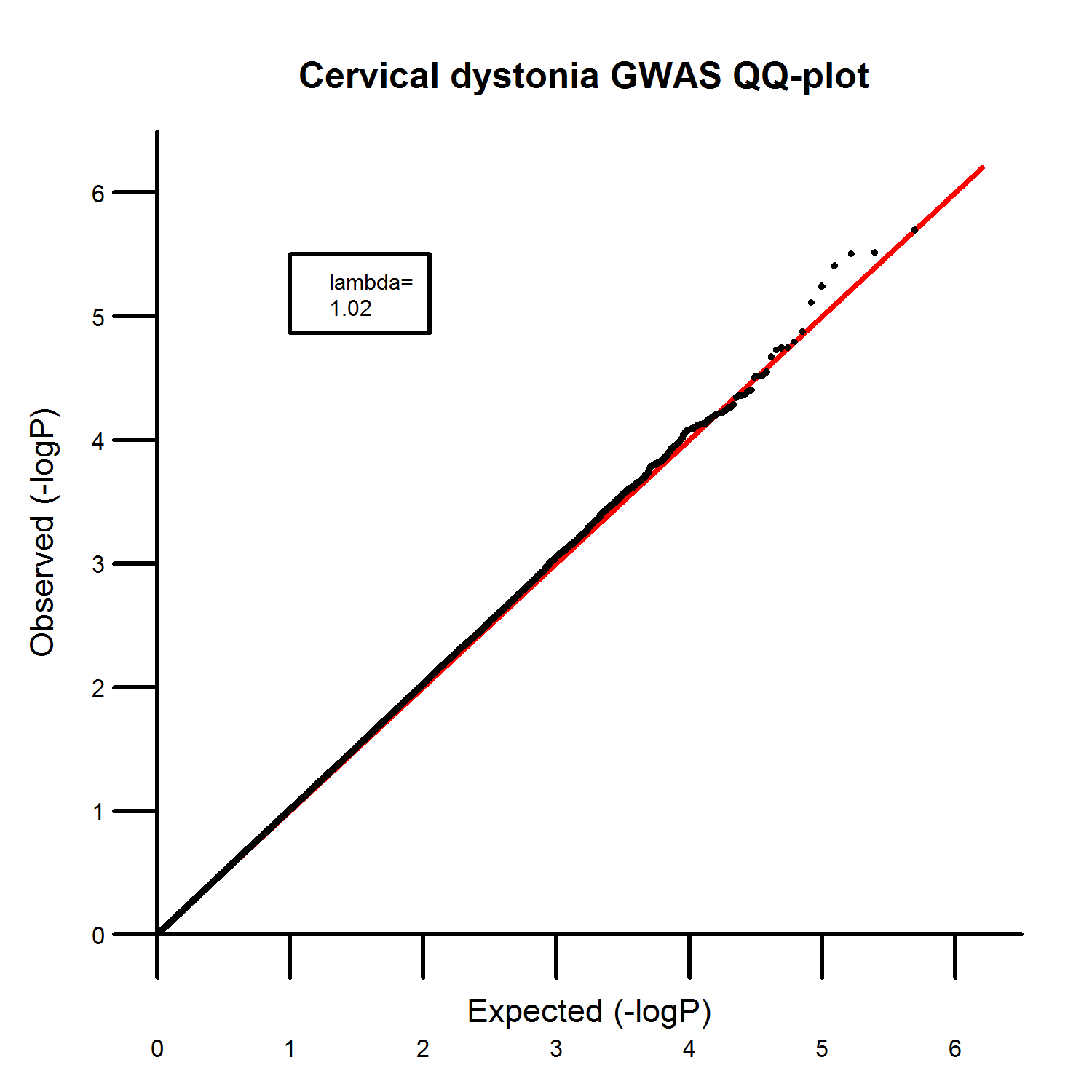

Supplement: Supplementary file 4 [file mds0029-0245-sd4.tif]

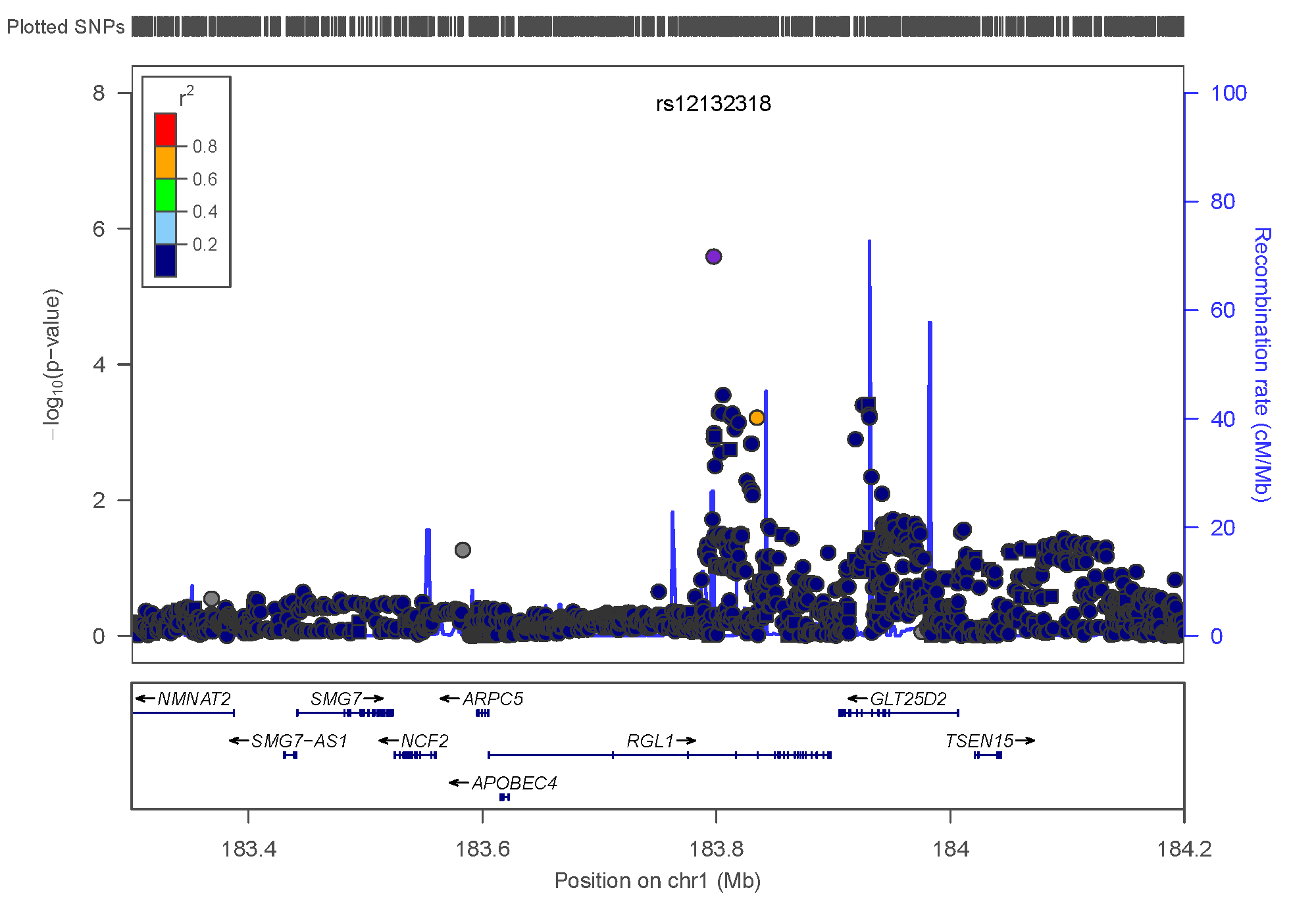

Supplement: Supplementary file 6 [file mds0029-0245-sd6.tif]

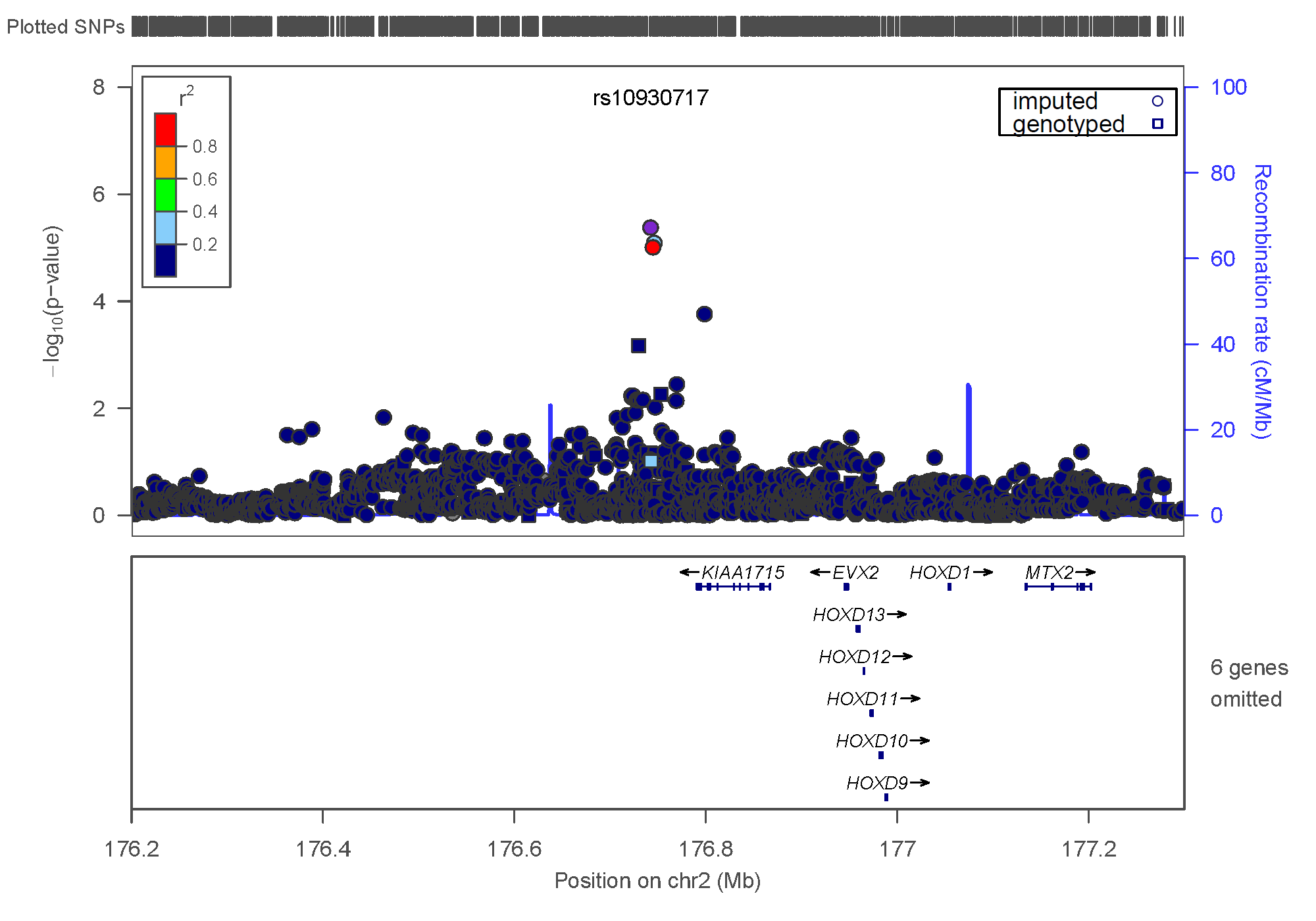

Supplement: Supplementary file 7 [file mds0029-0245-sd7.tif]

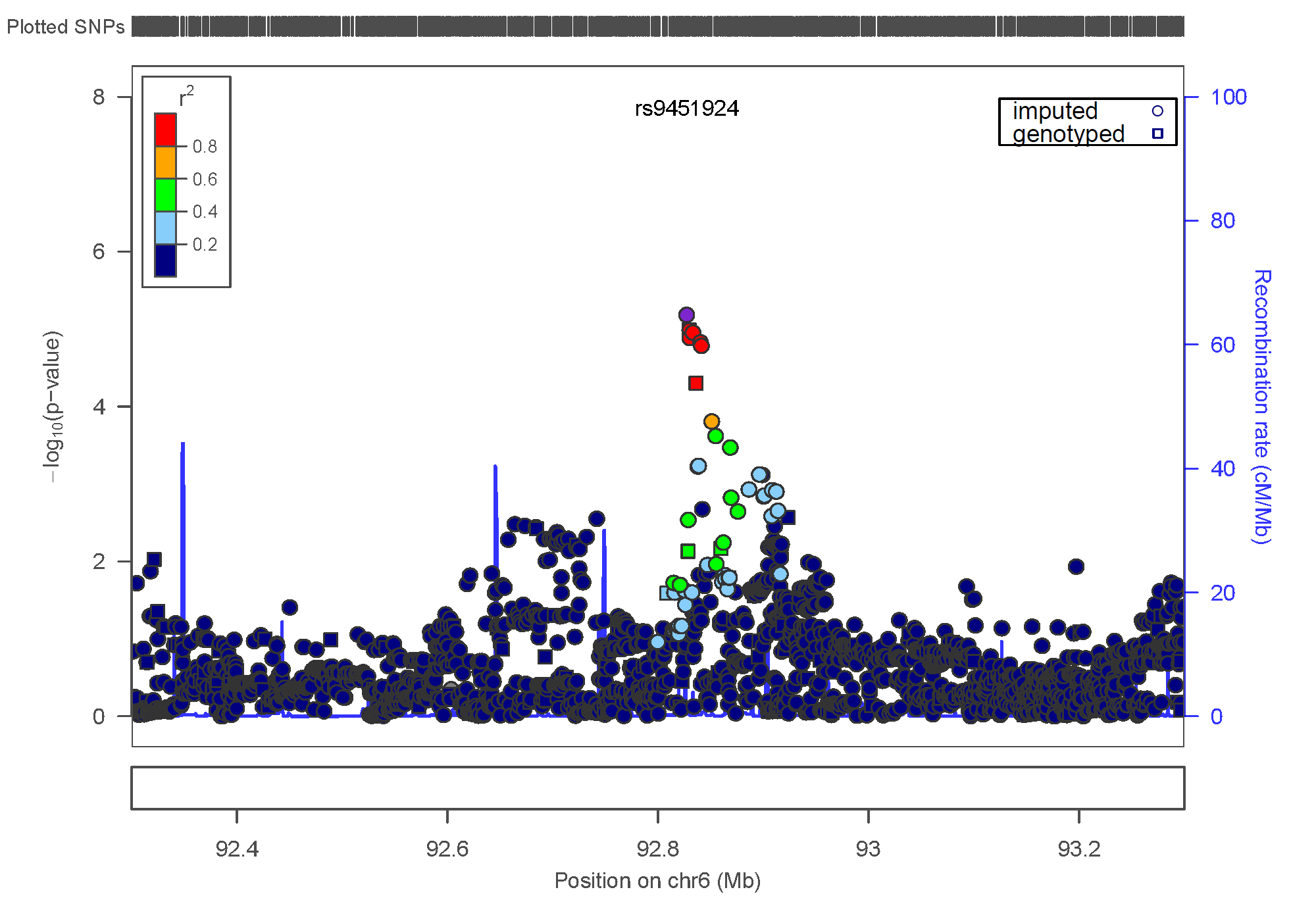

Supplement: Supplementary file 8 [file mds0029-0245-sd8.tif]

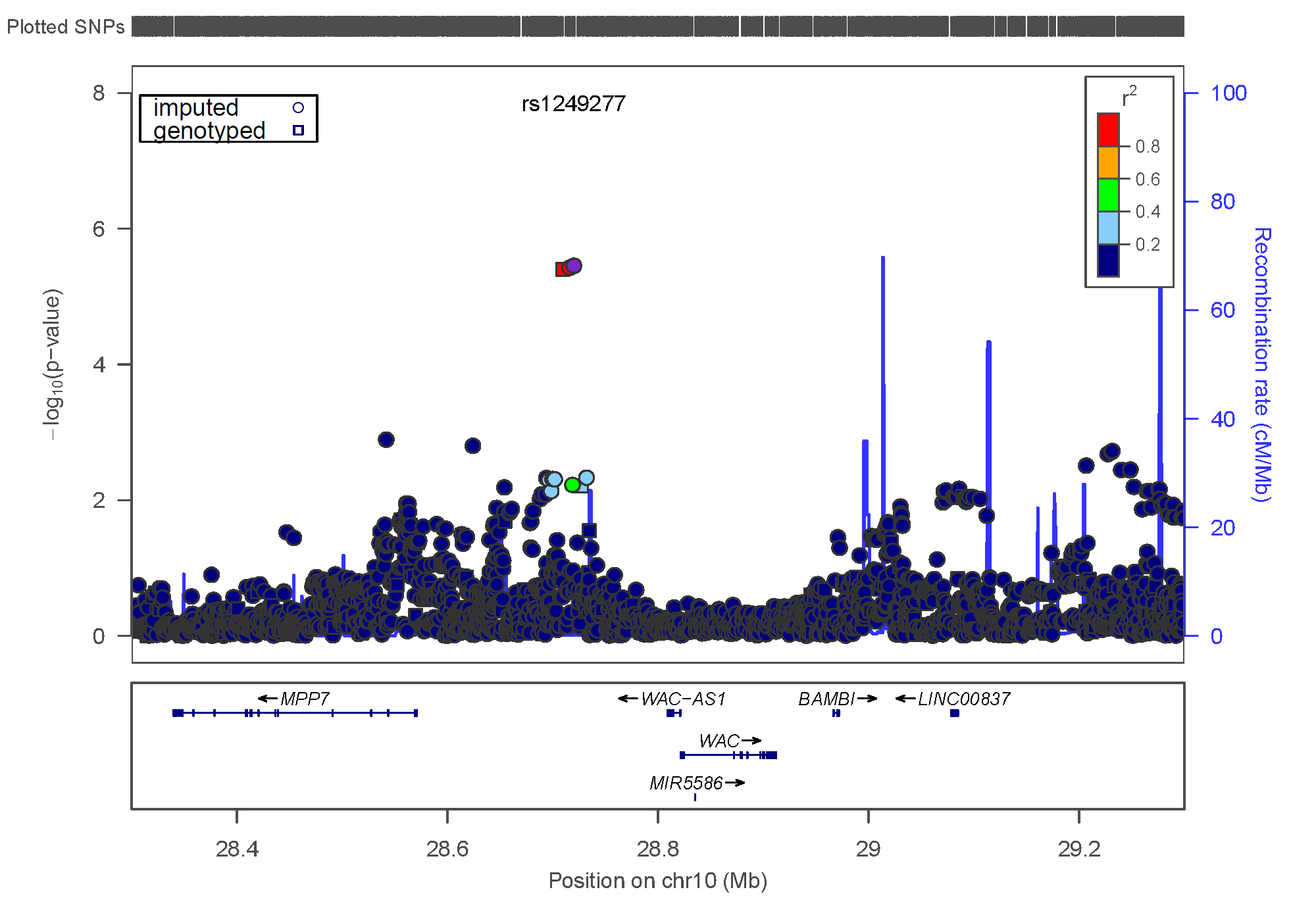

Supplement: Supplementary file 9 [file mds0029-0245-sd9.tif]

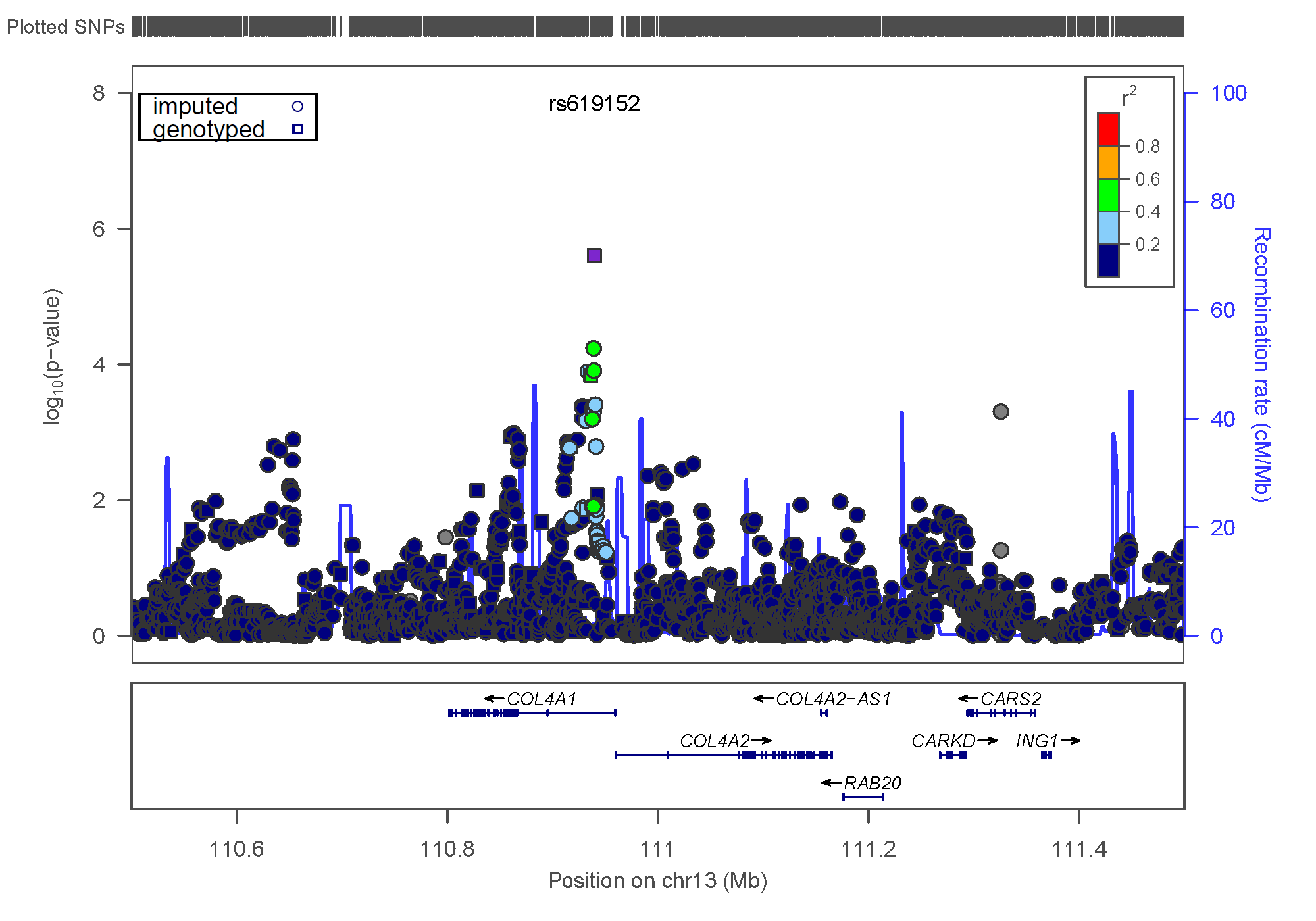

Supplement: Supplementary file 10 [file mds0029-0245-sd10.tif]

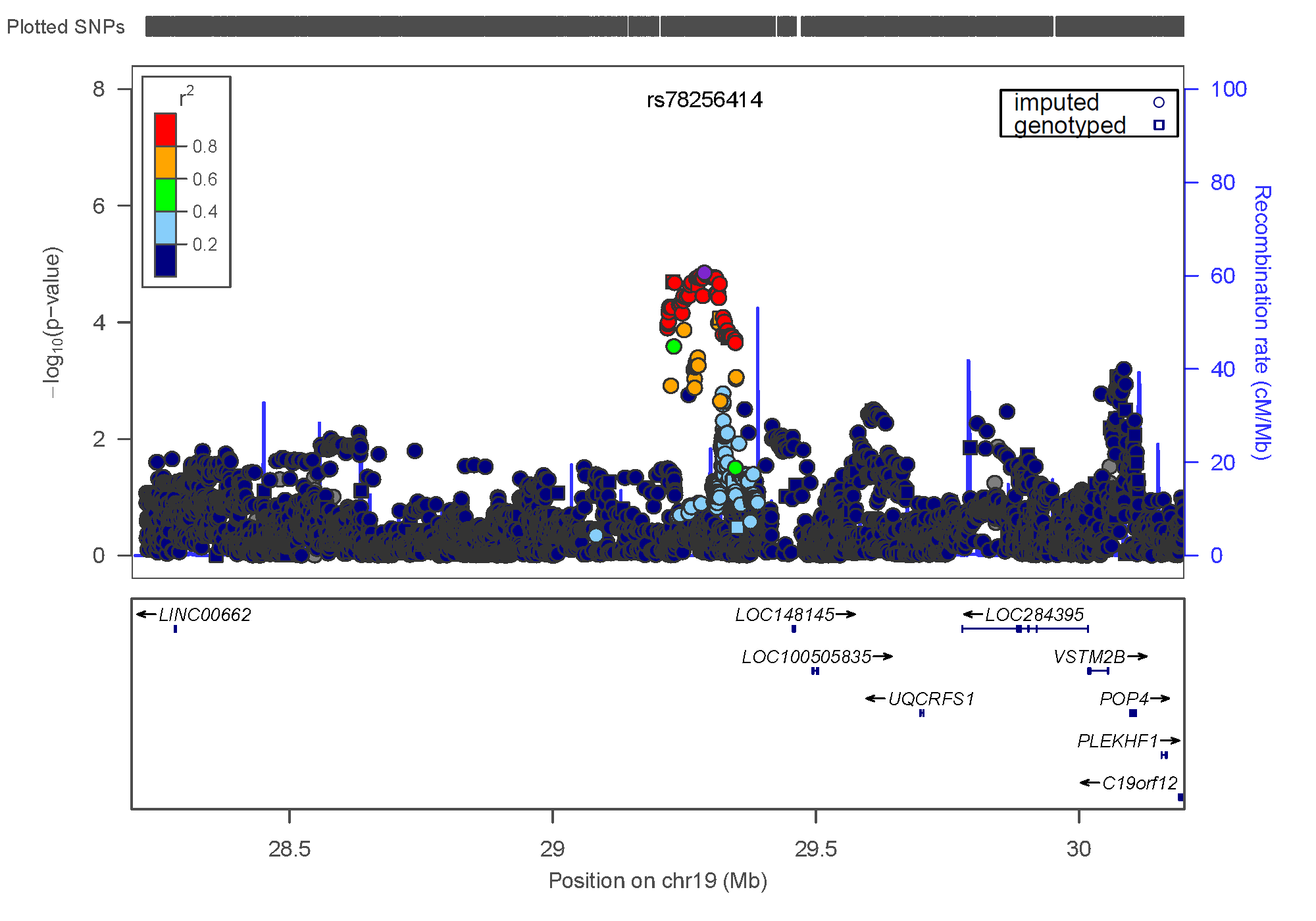

Supplement: Supplementary file 11 [file mds0029-0245-sd11.tif]

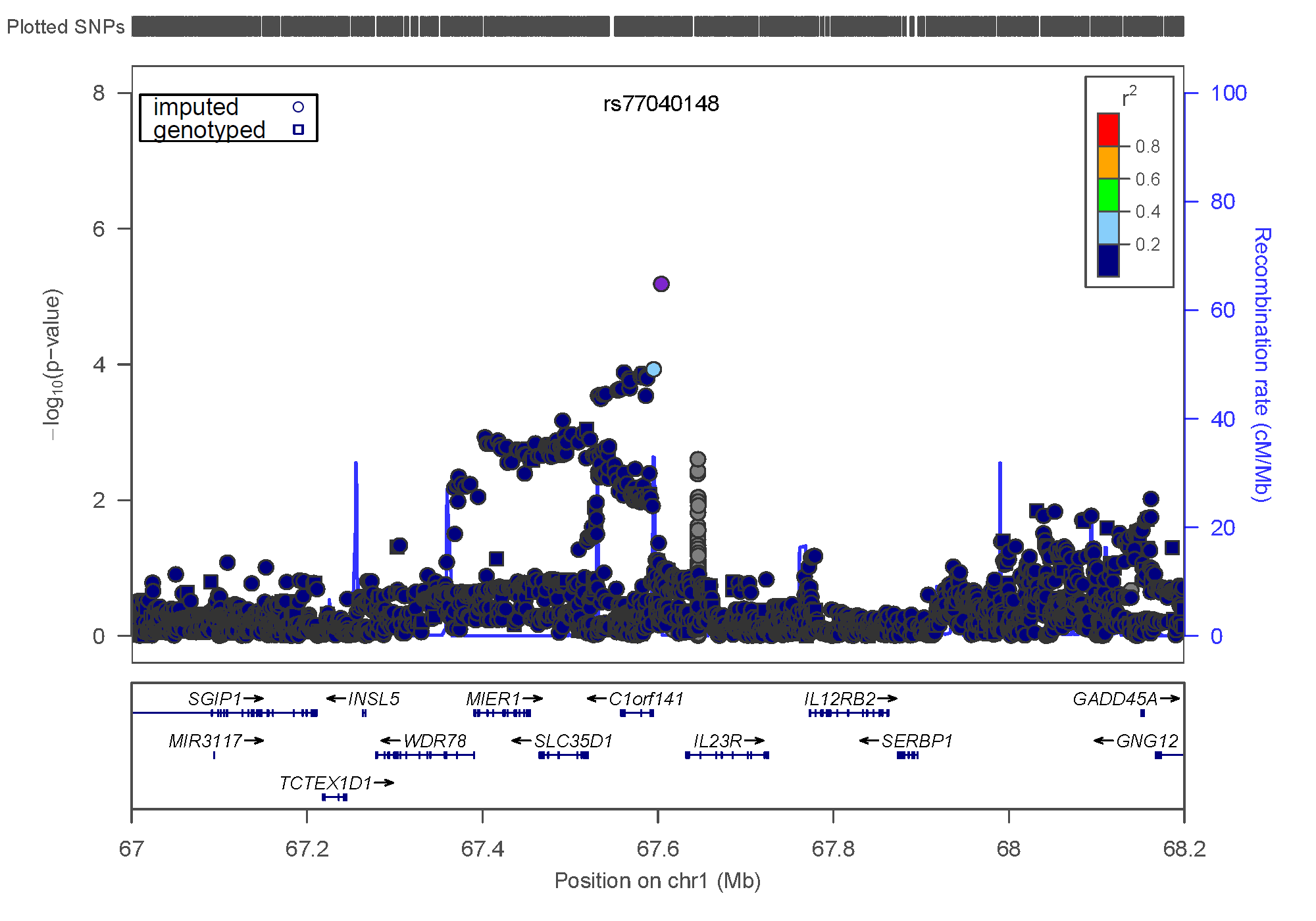

Supplement: Supplementary file 12 [file mds0029-0245-sd12.tif]

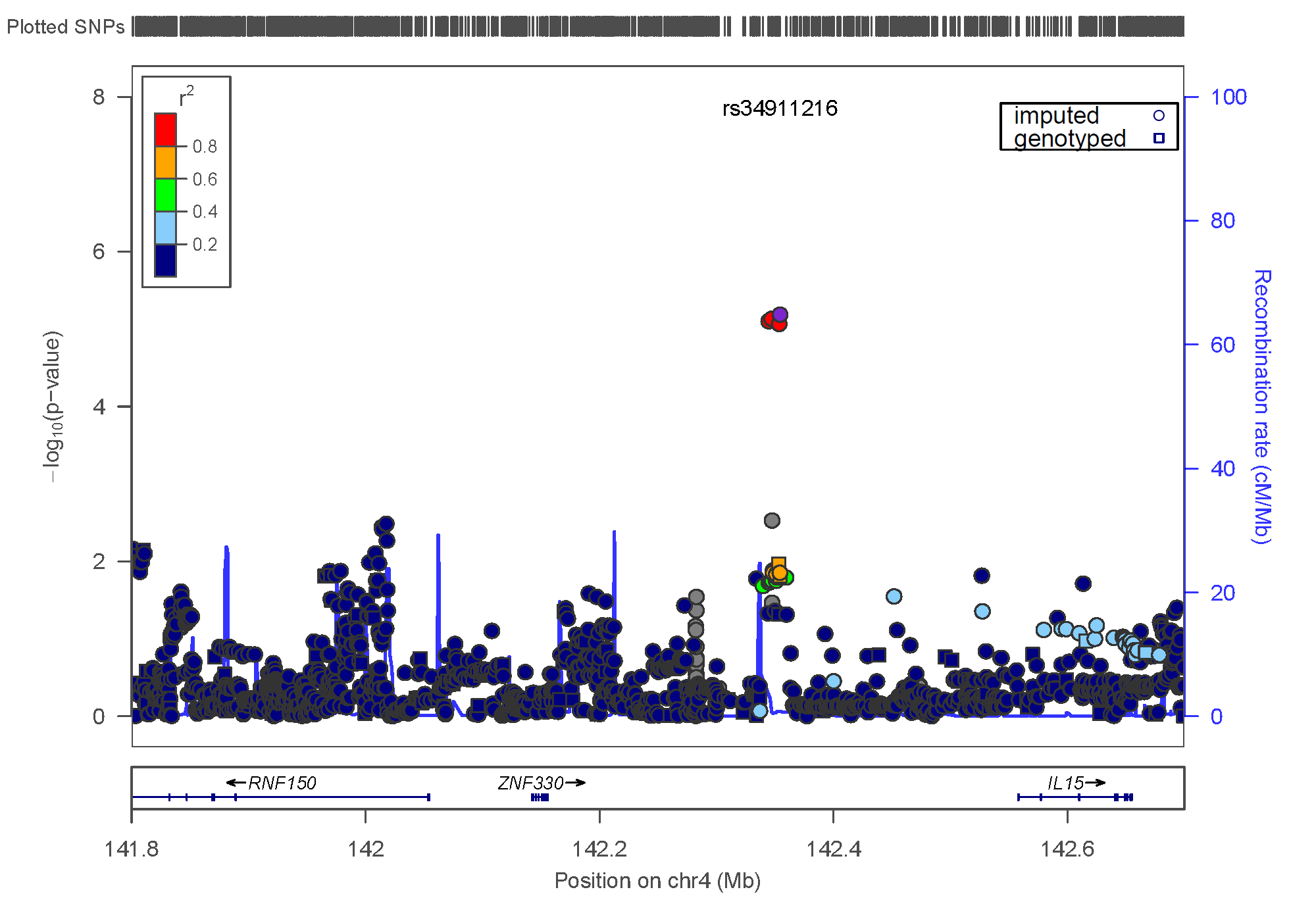

Supplement: Supplementary file 13 [file mds0029-0245-sd13.tif]

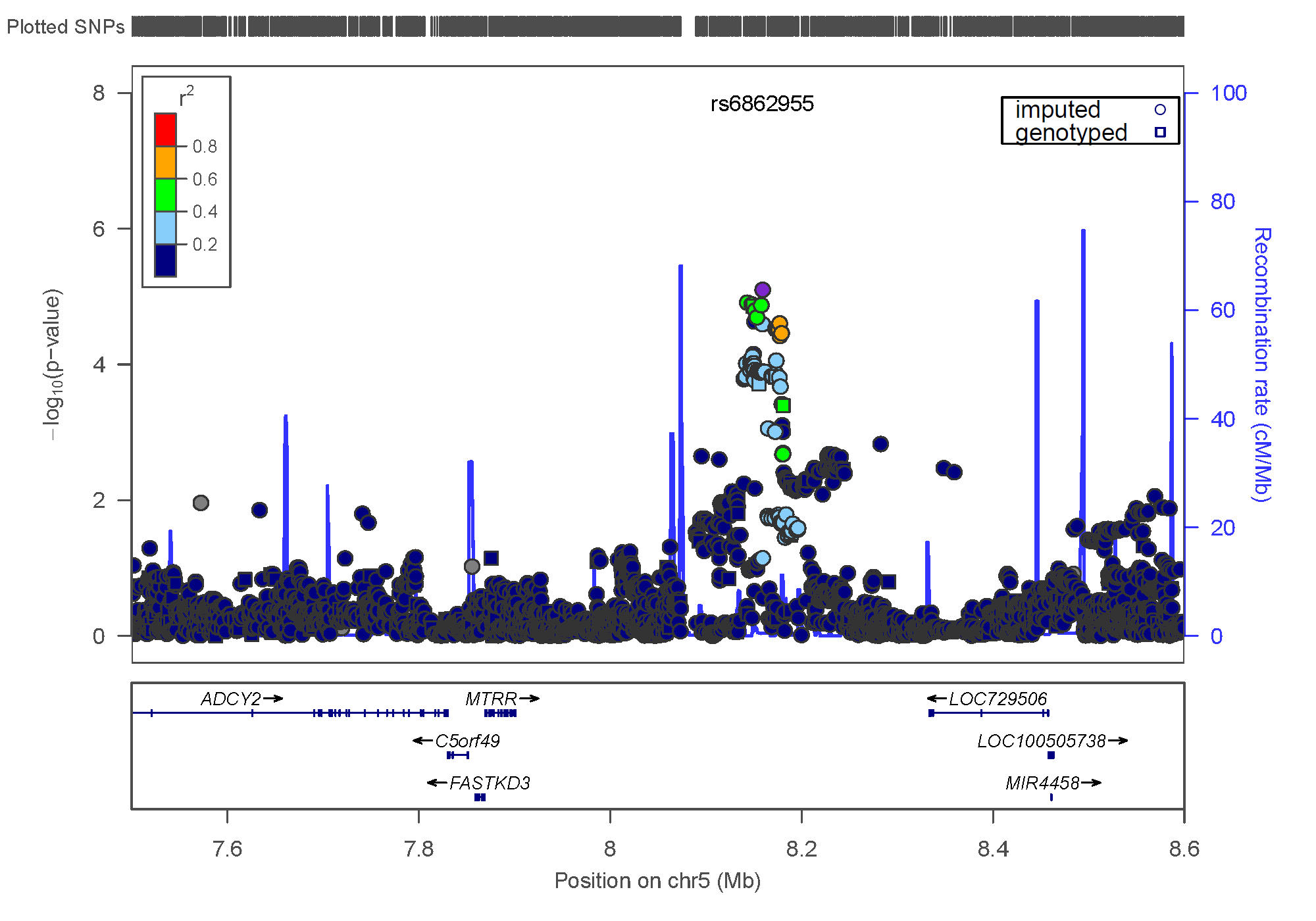

Supplement: Supplementary file 14 [file mds0029-0245-sd14.tif]

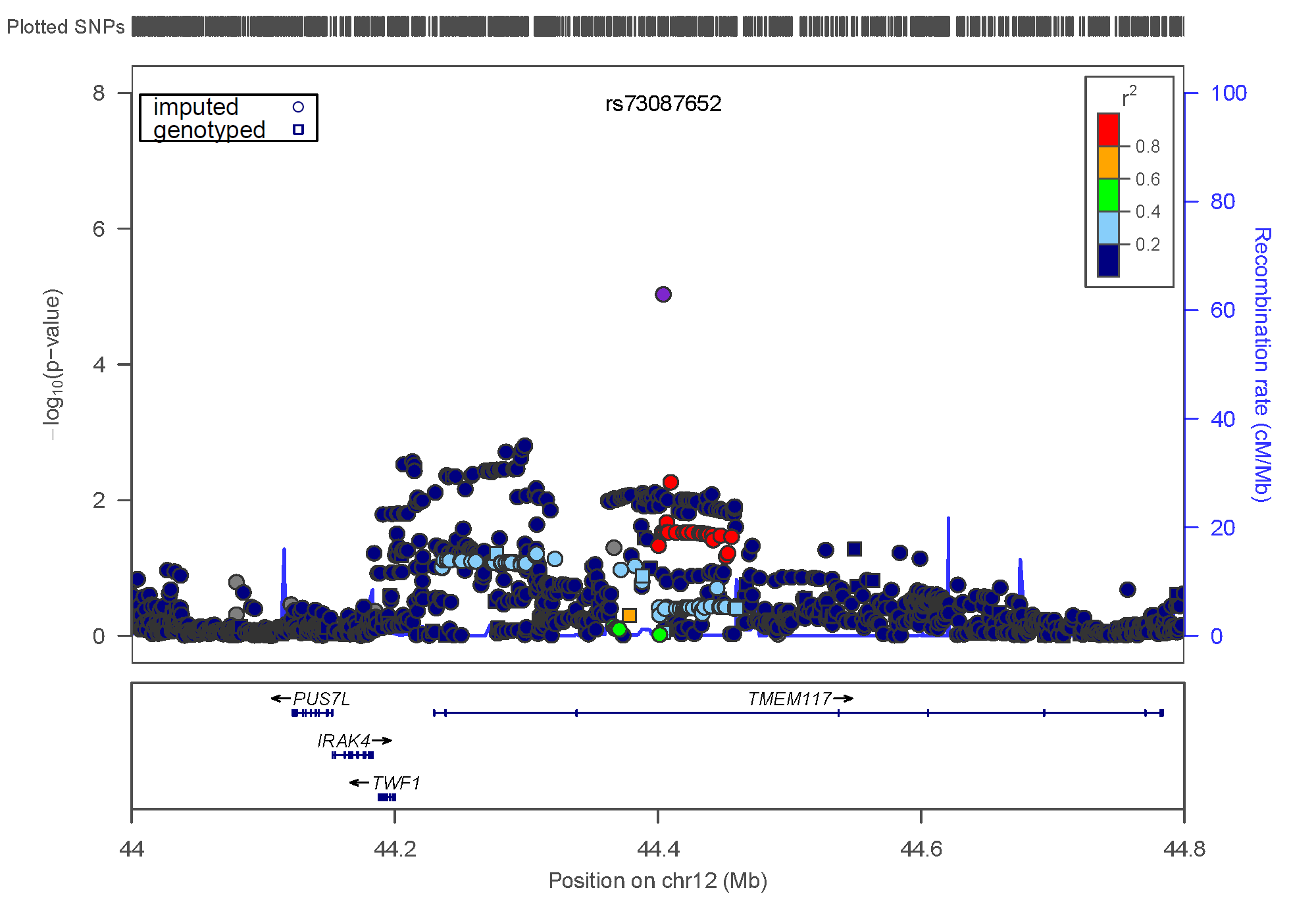

Supplement: Supplementary file 15 [file mds0029-0245-sd15.tif]

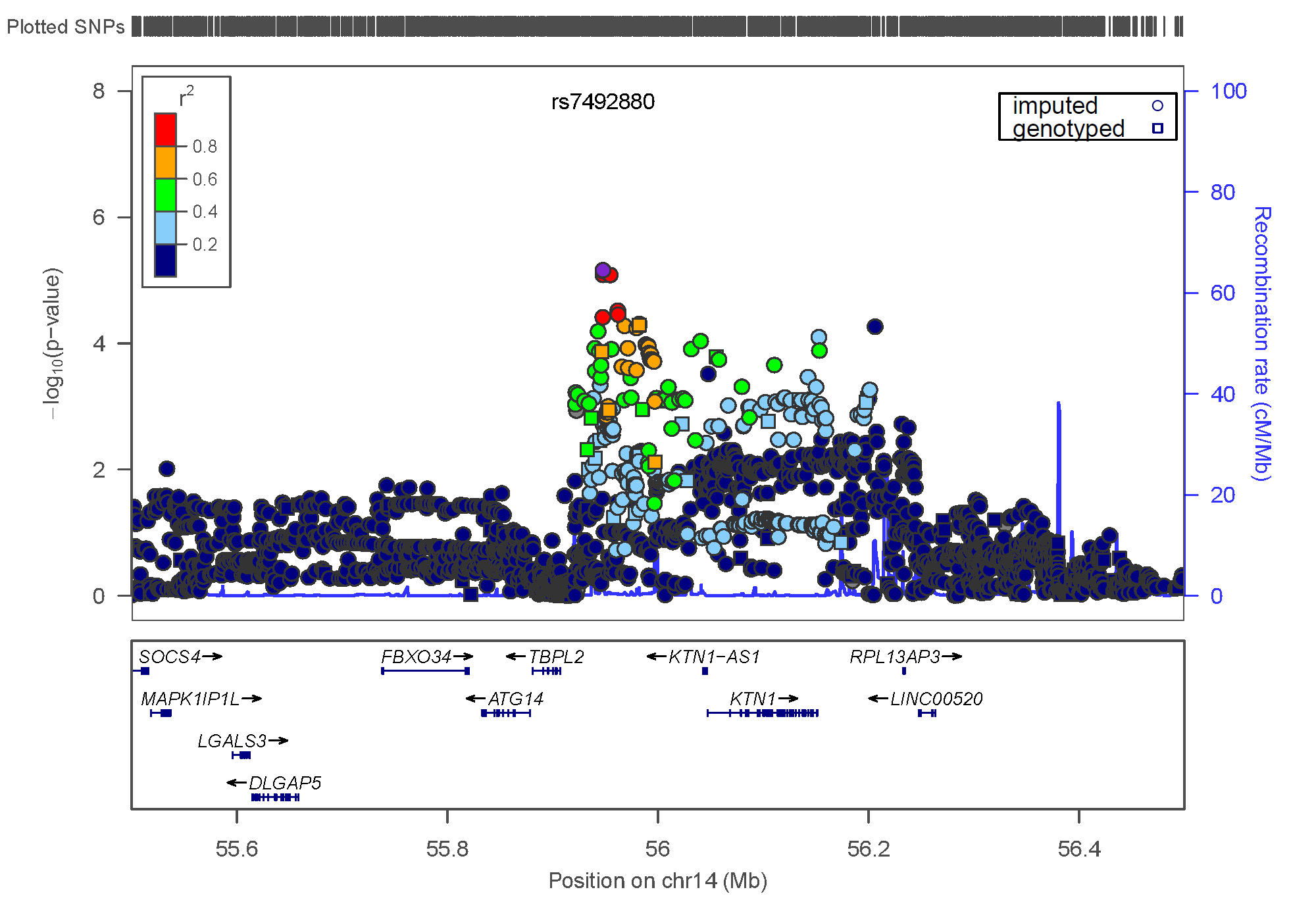

Supplement: Supplementary file 16 [file mds0029-0245-sd16.tif]
