# Supplementary material for: Genomewide association study in cervical dystonia demonstrates possible association with sodium leak channel
Source: Mov Disord. 2013 Nov 13;29(2):245–51. doi: 10.1002/mds.25732 (PMC4208301; doi:10.1002/mds.25732)
Supplement: Supplementary file 17 [file mds0029-0245-sd17.docx]

**S-Table 1** Cervical dystonia Case Sample exclusion breakdown

| Criteria for sample exclusion | Number Excluded |
| --- | --- |
| Call rate ( < 0.975 in genotyping array) | 10 |
| Gender discrepancy | 2 |
| Relatedness check (Duplication/relatedness, piHat > 0.125) | 2 |
| Population outlier (6SD outside mean of CEU TSI PLINK MDS axis 1 &2) | 7 |
