# Supplementary material for: Genomewide association study in cervical dystonia demonstrates possible association with sodium leak channel
Source: Mov Disord. 2013 Nov 13;29(2):245–51. doi: 10.1002/mds.25732 (PMC4208301; doi:10.1002/mds.25732)
Supplement: Supplementary file 18 [file mds0029-0245-sd18.docx]

**S-Table 2** Cervical Dystonia SNPs exclusion breakdown

| Criteria for SNP exclusion | Number excluded |
| --- | --- |
| SNPs not successfully genotyped or non-polymorphic SNPs (including cnv SNPs) | 36205 |
| failed QC in Genome Studio | 18922 |
| minor allele frequency < 0.01 | 31087 |
| Non-random missing due to haplotype (p < 1x10^-4^) | 1550 |
| Non-random missing due to phenotype (p < 1x10^-4^) | 2379 |
| Failed in Hardy-Weinberg equilibrium (p <1x10^-6^ in case) | 15 |
| total SNPs removed | 85427 |
| total SNPs remained after QC for cases | 534644 |
| Total SNPs in common after merged with WTCCC | 494025 |
