# Supplementary material for: Genomewide association study in cervical dystonia demonstrates possible association with sodium leak channel
Source: Mov Disord. 2013 Nov 13;29(2):245–51. doi: 10.1002/mds.25732 (PMC4208301; doi:10.1002/mds.25732)
Supplement: Supplementary file 19 [file mds0029-0245-sd19.docx]

**S-Table 3**

GWAS association top signals with P < 1x10^-5^ (The red labelled SNPs in Figure 1a)

| SNP | CHR | BP(hg19) | Minor | P | type | nearest genes |
| --- | --- | --- | --- | --- | --- | --- |
| rs9416795 | 10 | 28709550 | A | 2.00E-06 | intergenic | *MPP7 / WAC* |
| rs1338041 | 13 | 102058862 | G | 3.04E-06 | intron | *NALCN* |
| rs619152 | 13 | 110939497 | T | 3.10E-06 | inton | *COL4A1* |
| rs7249844 | 19 | 29228263 | C | 3.89E-06 | intergenic | *LOC100420587 / LOC100129507 ** |
| rs11672218 | 19 | 29275048 | A | 5.69E-06 | intergenic |  |
| rs4446534 | 6 | 92829488 | A | 7.73E-06 | intergenic | *RPL5P19* |

*Name based on NCBI SNP database
