# Supplementary material for: Genomewide association study in cervical dystonia demonstrates possible association with sodium leak channel
Source: Mov Disord. 2013 Nov 13;29(2):245–51. doi: 10.1002/mds.25732 (PMC4208301; doi:10.1002/mds.25732)
Supplement: Supplementary file 20 [file mds0029-0245-sd20.docx]

**S-Table 4 Post imputation SNPs with p < 1x 10-5**

**(The red labelled SNPs in Figure 1-b)**

| SNP | CHR | Position | Major Allele | Frequency of major allele | RSQR | EFFECT1 | OR | Std Error | Likelihood ratio ChiSq | P value of LR |
| --- | --- | --- | --- | --- | --- | --- | --- | --- | --- | --- |
| rs12132318 | 1 | 183797688 | T | 0.9376 | 0.3209 | -1.422 | 0.241 | 0.28 | 22.0992 | 2.59E-06 |
| rs77040148 | 1 | 67603833 | A | 0.9603 | 0.4003 | -1.387 | 0.25 | 0.276 | 20.3172 | 6.56E-06 |
| rs10930717 | 2 | 176742322 | G | 0.9524 | 0.9092 | -0.896 | 0.408 | 0.177 | 21.1596 | 4.23E-06 |
| rs13402361 | 2 | 158100907 | T | 0.6086 | 0.7562 | 0.553 | 1.738 | 0.128 | 19.8911 | 8.20E-06 |
| rs2166524 | 2 | 176744651 | A | 0.9537 | 0.9114 | -0.869 | 0.419 | 0.179 | 19.5332 | 9.89E-06 |
| rs2360969 | 2 | 208372996 | C | 0.5632 | 0.9998 | 0.46 | 1.584 | 0.106 | 19.6916 | 9.10E-06 |
| rs4972492 | 2 | 176746115 | T | 0.8769 | 0.8096 | -0.657 | 0.518 | 0.139 | 19.8901 | 8.20E-06 |
| rs13131103 | 4 | 142353633 | T | 0.8038 | 0.968 | 0.644 | 1.904 | 0.156 | 19.7953 | 8.62E-06 |
| rs34911216 | 4 | 142354352 | A | 0.8125 | 0.8809 | 0.699 | 2.011 | 0.167 | 20.3236 | 6.54E-06 |
| rs34980774 | 4 | 142347175 | A | 0.7988 | 0.9853 | 0.637 | 1.891 | 0.153 | 20.0863 | 7.40E-06 |
| rs35671858 | 4 | 142344663 | A | 0.7986 | 0.9747 | 0.638 | 1.892 | 0.154 | 19.9508 | 7.95E-06 |
| rs6862955 | 5 | 8159422 | C | 0.6263 | 0.6714 | 0.589 | 1.802 | 0.136 | 19.9372 | 8.00E-06 |
| rs9451924 | 6 | 92827234 | G | 0.6605 | 0.967 | -0.461 | 0.631 | 0.101 | 20.3105 | 6.58E-06 |
| rs1249277 | 10 | 28720076 | G | 0.8497 | 0.9894 | -0.574 | 0.563 | 0.118 | 21.5017 | 3.54E-06 |
| rs1249281 | 10 | 28716177 | G | 0.8505 | 0.9956 | -0.572 | 0.564 | 0.118 | 21.3706 | 3.79E-06 |
| rs9416795 | 10 | 28709550 | G | 0.8508 | 0.9998 | -0.57 | 0.565 | 0.118 | 21.2877 | 3.95E-06 |
| rs35875350 | 11 | 48230490 | G | 0.9433 | 0.9333 | 1.513 | 4.538 | 0.421 | 20.9828 | 4.63E-06 |
| rs550376 | 11 | 70495262 | A | 0.6182 | 0.6243 | -0.566 | 0.568 | 0.127 | 19.6733 | 9.19E-06 |
| rs551260 | 11 | 70501393 | T | 0.6348 | 0.6558 | -0.567 | 0.567 | 0.124 | 20.5395 | 5.84E-06 |
| rs67863238 | 11 | 48267856 | G | 0.9439 | 0.8808 | 1.608 | 4.994 | 0.442 | 21.7354 | 3.13E-06 |
| rs73087652 | 12 | 44404068 | T | 0.9616 | 0.9926 | 1.988 | 7.297 | 0.645 | 19.6425 | 9.34E-06 |
| rs1338041 | 13 | 102058862 | A | 0.6597 | 0.9955 | -0.48 | 0.619 | 0.1 | 22.4931 | 2.11E-06 |
| rs1338051 | 13 | 102062341 | G | 0.6595 | 0.9932 | -0.481 | 0.618 | 0.1 | 22.5497 | 2.05E-06 |
| rs3916908 | 13 | 102058054 | A | 0.6602 | 0.9949 | -0.477 | 0.621 | 0.1 | 22.1374 | 2.54E-06 |
| rs619152 | 13 | 110939497 | G | 0.6437 | 0.9328 | -0.489 | 0.614 | 0.103 | 22.1724 | 2.49E-06 |
| rs61973742 | 13 | 102083273 | A | 0.9384 | 0.8881 | 1.579 | 4.848 | 0.415 | 23.9755 | 9.76E-07 |
| rs80239252 | 13 | 74877891 | A | 0.9694 | 0.6417 | 3.134 | 22.964 | 1.064 | 19.6872 | 9.12E-06 |
| rs9518384 | 13 | 102059871 | C | 0.6597 | 0.9951 | -0.48 | 0.619 | 0.1 | 22.5246 | 2.08E-06 |
| rs9518385 | 13 | 102060280 | A | 0.6597 | 0.9947 | -0.48 | 0.618 | 0.1 | 22.5411 | 2.06E-06 |
| rs2341882 | 14 | 55954640 | T | 0.5538 | 0.9459 | -0.459 | 0.632 | 0.104 | 19.8685 | 8.30E-06 |
| rs2341900 | 14 | 55947850 | C | 0.5502 | 0.9542 | -0.46 | 0.631 | 0.103 | 20.1458 | 7.18E-06 |
| rs7492716 | 14 | 55947821 | C | 0.5534 | 0.9438 | -0.461 | 0.631 | 0.104 | 19.8735 | 8.27E-06 |
| rs7492880 | 14 | 55947784 | T | 0.5846 | 0.8662 | -0.488 | 0.614 | 0.109 | 20.2059 | 6.95E-06 |
