# Supplementary material for: Genomewide association study in cervical dystonia demonstrates possible association with sodium leak channel
Source: Mov Disord. 2013 Nov 13;29(2):245–51. doi: 10.1002/mds.25732 (PMC4208301; doi:10.1002/mds.25732)
Supplement: Supplementary file 21 [file mds0029-0245-sd21.docx]

**S-table 5 Post-imputation SNPs with p < 1x 10^-3^ in Chr 11 *OR4X1* region**

| SNP | CHR | Position | Major Allele | Frequency of major allele | RSQR | EFFECT1 | OR | P of likelihood ratio |
| --- | --- | --- | --- | --- | --- | --- | --- | --- |
| rs35800856 | 11 | 48133397 | G | 0.939 | 0.8368 | 0.947 | 2.578 | 0.000929 |
| rs12800804 | 11 | 48178598 | G | 0.889 | 0.9167 | 0.635 | 1.886 | 0.000849 |
| rs12809012 | 11 | 48188872 | G | 0.9346 | 0.9223 | 1.151 | 3.161 | 5.63E-05 |
| rs12798703 | 11 | 48190777 | G | 0.9377 | 0.9996 | 1.155 | 3.173 | 4.38E-05 |
| rs59168438 | 11 | 48196641 | A | 0.8815 | 0.9661 | 0.618 | 1.856 | 0.000614 |
| rs12803468 | 11 | 48196964 | C | 0.9377 | 0.9875 | 1.163 | 3.2 | 4.34E-05 |
| rs2018610 | 11 | 48198705 | T | 0.8815 | 0.9663 | 0.619 | 1.857 | 0.000604 |
| rs35127500 | 11 | 48199847 | G | 0.9394 | 0.9506 | 1.218 | 3.38 | 3.65E-05 |
| rs9633889 | 11 | 48203984 | C | 0.8773 | 0.9363 | 0.629 | 1.875 | 0.00051 |
| rs11039567 | 11 | 48205074 | T | 0.8792 | 0.9558 | 0.627 | 1.872 | 0.000505 |
| rs7108495 | 11 | 48211281 | A | 0.8806 | 0.9702 | 0.628 | 1.873 | 0.000486 |
| rs2202248 | 11 | 48211435 | T | 0.8766 | 0.9356 | 0.628 | 1.873 | 0.000507 |
| rs4417227 | 11 | 48212666 | C | 0.8791 | 0.9587 | 0.629 | 1.876 | 0.000476 |
| chr11:48212741 | 11 | 48212741 | T | 0.937 | 0.9862 | 1.168 | 3.214 | 3.84E-05 |
| rs12223266 | 11 | 48214982 | G | 0.8815 | 0.996 | 0.632 | 1.882 | 0.000399 |
| rs7111194 | 11 | 48214992 | C | 0.8815 | 0.9963 | 0.632 | 1.882 | 0.000397 |
| rs1910363 | 11 | 48216268 | C | 0.8832 | 0.9766 | 0.631 | 1.88 | 0.000485 |
| rs35492140 | 11 | 48217842 | C | 0.9373 | 0.9935 | 1.164 | 3.202 | 3.88E-05 |
| rs61916993 | 11 | 48219648 | G | 0.8781 | 0.9571 | 0.638 | 1.893 | 0.000387 |
| rs7396453 | 11 | 48220378 | T | 0.8806 | 0.9778 | 0.633 | 1.883 | 0.000418 |
| rs11500493 | 11 | 48222224 | G | 0.882 | 0.9564 | 0.619 | 1.857 | 0.000645 |
| rs11039583 | 11 | 48222605 | A | 0.88 | 0.9658 | 0.633 | 1.884 | 0.000436 |
| rs34207686 | 11 | 48224387 | A | 0.9384 | 0.9747 | 1.186 | 3.274 | 3.84E-05 |
| rs10838827 | 11 | 48225889 | C | 0.8702 | 0.8981 | 0.592 | 1.807 | 0.000874 |
| rs12802303 | 11 | 48226477 | C | 0.9373 | 0.9946 | 1.161 | 3.194 | 3.91E-05 |
| rs12278241 | 11 | 48228595 | C | 0.8796 | 0.9565 | 0.633 | 1.883 | 0.000458 |
| rs2174128 | 11 | 48229742 | G | 0.8796 | 0.9552 | 0.633 | 1.883 | 0.00046 |
| rs35838205 | 11 | 48230035 | A | 0.9373 | 0.997 | 1.161 | 3.193 | 3.88E-05 |
| rs35875350 | 11 | 48230490 | G | 0.9433 | 0.9333 | 1.513 | 4.538 | 4.63E-06 |
| rs67356160 | 11 | 48231182 | A | 0.9421 | 0.9344 | 1.207 | 3.344 | 6.05E-05 |
| rs10769328 | 11 | 48232972 | C | 0.8795 | 0.9544 | 0.633 | 1.882 | 0.000462 |
| rs10742841 | 11 | 48234206 | T | 0.9374 | 0.9982 | 1.161 | 3.193 | 3.87E-05 |
| rs67253125 | 11 | 48234230 | A | 0.9371 | 0.9947 | 1.164 | 3.203 | 3.75E-05 |
| rs905480 | 11 | 48237461 | G | 0.9374 | 0.9996 | 1.16 | 3.192 | 3.88E-05 |
| rs12292056 | 11 | 48239071 | C | 0.9374 | 1 | 1.16 | 3.191 | 3.88E-05 |
| rs34070700 | 11 | 48240177 | T | 0.9474 | 0.861 | 1.327 | 3.771 | 6.02E-05 |
| rs67938441 | 11 | 48240278 | C | 0.9378 | 0.9934 | 1.163 | 3.201 | 4.1E-05 |
| rs67630889 | 11 | 48245720 | T | 0.9364 | 0.9785 | 1.175 | 3.237 | 3.51E-05 |
| rs12271090 | 11 | 48247600 | G | 0.938 | 0.8638 | 1.313 | 3.717 | 2.09E-05 |
| rs113294115 | 11 | 48252416 | C | 0.9358 | 0.9743 | 1.189 | 3.283 | 2.86E-05 |
| rs112695799 | 11 | 48255734 | T | 0.9654 | 0.659 | 1.839 | 6.288 | 0.000173 |
| rs12797239 | 11 | 48255953 | G | 0.9363 | 0.9693 | 1.188 | 3.282 | 3.11E-05 |
| rs7113888 | 11 | 48260035 | C | 0.9353 | 0.9639 | 1.196 | 3.308 | 2.71E-05 |
| rs67863238 | 11 | 48267856 | G | 0.9439 | 0.8808 | 1.608 | 4.994 | 3.13E-06 |
| rs1910360 | 11 | 48270533 | A | 0.9336 | 0.9456 | 1.21 | 3.352 | 2.21E-05 |
| rs11039614 | 11 | 48271175 | C | 0.9362 | 0.9847 | 1.177 | 3.244 | 3.2E-05 |
| rs11039615 | 11 | 48271714 | A | 0.9362 | 0.9849 | 1.177 | 3.245 | 3.2E-05 |
| rs12278363 | 11 | 48272341 | T | 0.9362 | 0.985 | 1.177 | 3.244 | 3.21E-05 |
| rs66949996 | 11 | 48275112 | T | 0.9368 | 0.985 | 1.176 | 3.242 | 3.41E-05 |
| rs59173570 | 11 | 48278913 | A | 0.9365 | 0.9914 | 1.174 | 3.234 | 3.26E-05 |
| rs34290179 | 11 | 48283811 | C | 0.9365 | 0.9937 | 1.172 | 3.227 | 3.3E-05 |
| rs12807125 | 11 | 48283979 | C | 0.9366 | 0.9938 | 1.172 | 3.227 | 3.3E-05 |
| rs12788290 | 11 | 48284259 | A | 0.9396 | 0.9554 | 1.214 | 3.367 | 3.68E-05 |
| rs67245906 | 11 | 48285361 | A | 0.9382 | 0.9763 | 1.195 | 3.304 | 3.37E-05 |
| rs12799818 | 11 | 48287931 | T | 0.9368 | 1 | 1.167 | 3.211 | 3.42E-05 |
| rs12283746 | 11 | 48289963 | C | 0.9341 | 0.9589 | 1.197 | 3.312 | 2.44E-05 |
| rs67624356 | 11 | 48292620 | C | 0.9387 | 0.9712 | 1.188 | 3.281 | 3.99E-05 |
| rs7484031 | 11 | 48293468 | G | 0.9368 | 1 | 1.168 | 3.216 | 3.36E-05 |
| rs7481374 | 11 | 48293530 | T | 0.9368 | 1 | 1.168 | 3.216 | 3.36E-05 |
| rs12806319 | 11 | 48297297 | T | 0.9364 | 0.9774 | 1.179 | 3.253 | 3.31E-05 |
| rs12795303 | 11 | 48299748 | C | 0.9363 | 0.9755 | 1.181 | 3.256 | 3.3E-05 |
| rs12795320 | 11 | 48299812 | A | 0.9358 | 0.9674 | 1.189 | 3.285 | 3.02E-05 |
| rs12296067 | 11 | 48301855 | C | 0.9357 | 0.9591 | 1.194 | 3.299 | 3.01E-05 |
| rs7946363 | 11 | 48308303 | G | 0.9345 | 0.5527 | 1.565 | 4.781 | 2.28E-05 |
| rs11530190 | 11 | 48309157 | C | 0.9436 | 0.5388 | 1.67 | 5.312 | 4.95E-05 |
| rs7106486 | 11 | 48310553 | G | 0.934 | 0.8485 | 1.247 | 3.479 | 3.17E-05 |
| rs35077153 | 11 | 48310636 | G | 0.7717 | 0.7069 | -0.445 | 0.641 | 0.000864 |
| rs12788787 | 11 | 48314483 | C | 0.938 | 0.8105 | 1.314 | 3.721 | 3.2E-05 |
| rs34579705 | 11 | 48314728 | G | 0.9349 | 0.8449 | 1.266 | 3.547 | 2.9E-05 |
| rs7124493 | 11 | 48315258 | C | 0.9349 | 0.8448 | 1.266 | 3.546 | 2.91E-05 |
| rs11530205 | 11 | 48316815 | T | 0.9349 | 0.8354 | 1.274 | 3.576 | 2.87E-05 |
| rs10838866 | 11 | 48318362 | A | 0.9231 | 0.4188 | 1.316 | 3.73 | 0.000192 |
| rs66954292 | 11 | 48319692 | A | 0.935 | 0.8311 | 1.278 | 3.589 | 2.84E-05 |
| rs66828634 | 11 | 48321604 | T | 0.9345 | 0.8232 | 1.282 | 3.605 | 2.75E-05 |
| rs66531366 | 11 | 48322273 | C | 0.936 | 0.8033 | 1.311 | 3.712 | 2.76E-05 |
| rs12798215 | 11 | 48324997 | G | 0.9413 | 0.7428 | 1.437 | 4.209 | 2.47E-05 |
| rs7129548 | 11 | 48326619 | C | 0.9352 | 0.8122 | 1.298 | 3.661 | 2.7E-05 |
| rs34705503 | 11 | 48330198 | C | 0.9353 | 0.8106 | 1.3 | 3.668 | 2.69E-05 |
| rs1905284 | 11 | 48331739 | T | 0.9402 | 0.7535 | 1.393 | 4.029 | 2.9E-05 |
| rs7949550 | 11 | 48338856 | C | 0.9385 | 0.5593 | 1.594 | 4.923 | 2.99E-05 |
