# Supplementary material for: Genomewide association study in cervical dystonia demonstrates possible association with sodium leak channel
Source: Mov Disord. 2013 Nov 13;29(2):245–51. doi: 10.1002/mds.25732 (PMC4208301; doi:10.1002/mds.25732)
Supplement: Supplementary file 22 [file mds0029-0245-sd22.docx]

**S-table 6 Post-imputation SNPs with p < 1x 10^-3^ in Chr 11 *NALCN* region**

| SNP | CHR | Position | Major Allele | Frequency of major allele | RSQR | EFFECT1 | OR | P of likelihood ratio |
| --- | --- | --- | --- | --- | --- | --- | --- | --- |
| rs75927109 | 13 | 102041995 | G | 0.9526 | 0.9555 | 1.164 | 3.204 | 0.000339 |
| rs79694122 | 13 | 102041996 | G | 0.9526 | 0.9556 | 1.164 | 3.204 | 0.000338 |
| rs4772377 | 13 | 102049736 | C | 0.9506 | 0.9684 | 1.035 | 2.815 | 0.000783 |
| rs10508066 | 13 | 102055074 | C | 0.9501 | 0.9832 | 1.363 | 3.909 | 3.83E-05 |
| rs3916908 | 13 | 102058054 | A | 0.6602 | 0.9949 | -0.477 | 0.621 | 2.54E-06 |
| chr13:102058564 | 13 | 102058564 | C | 0.9504 | 0.9943 | 1.356 | 3.882 | 3.92E-05 |
| rs1338041 | 13 | 102058862 | A | 0.6597 | 0.9955 | -0.48 | 0.619 | 2.11E-06 |
| rs9518384 | 13 | 102059871 | C | 0.6597 | 0.9951 | -0.48 | 0.619 | 2.08E-06 |
| rs1572590 | 13 | 102060171 | T | 0.9503 | 0.9928 | 1.359 | 3.893 | 3.78E-05 |
| rs9518385 | 13 | 102060280 | A | 0.6597 | 0.9947 | -0.48 | 0.618 | 2.06E-06 |
| rs1338051 | 13 | 102062341 | G | 0.6595 | 0.9932 | -0.481 | 0.618 | 2.05E-06 |
| rs9585688 | 13 | 102065033 | C | 0.9497 | 0.9881 | 1.249 | 3.489 | 8.74E-05 |
| rs56406871 | 13 | 102075102 | G | 0.7618 | 0.9514 | -0.388 | 0.678 | 0.00068 |
| rs59318124 | 13 | 102081041 | C | 0.9461 | 0.9794 | 1.399 | 4.051 | 1.36E-05 |
| rs61973742 | 13 | 102083273 | A | 0.9384 | 0.8881 | 1.579 | 4.848 | 9.76E-07 |
| rs4772382 | 13 | 102087428 | T | 0.9462 | 0.9766 | 1.397 | 4.045 | 1.42E-05 |
| rs34716843 | 13 | 102090037 | G | 0.9624 | 0.9644 | 1.434 | 4.194 | 0.000247 |
| rs78657061 | 13 | 102090219 | G | 0.9644 | 0.9275 | 1.793 | 6.005 | 5.13E-05 |
| rs55736841 | 13 | 102092192 | A | 0.9584 | 0.7804 | 1.447 | 4.249 | 0.000249 |
| rs9518411 | 13 | 102092504 | C | 0.7583 | 0.9688 | -0.396 | 0.673 | 0.000437 |
| rs6491618 | 13 | 102094484 | G | 0.7928 | 0.9277 | 0.575 | 1.778 | 4.54E-05 |
| rs4325410 | 13 | 102094957 | A | 0.9508 | 0.8986 | 1.393 | 4.025 | 5.78E-05 |
| rs76933615 | 13 | 102095304 | C | 0.9624 | 0.9614 | 1.433 | 4.193 | 0.00025 |
| rs1106123 | 13 | 102095862 | C | 0.8076 | 0.9684 | 0.597 | 1.817 | 3.15E-05 |
| rs1106125 | 13 | 102096112 | T | 0.7423 | 0.7346 | 0.474 | 1.606 | 0.000888 |
| rs7985216 | 13 | 102098107 | A | 0.9504 | 0.8945 | 1.394 | 4.03 | 5.49E-05 |
| rs4772385 | 13 | 102111521 | T | 0.9403 | 0.8286 | 1.418 | 4.13 | 1.51E-05 |
| rs7984480 | 13 | 102116884 | A | 0.65 | 0.584 | -0.549 | 0.577 | 2.49E-05 |
| rs9518419 | 13 | 102117812 | A | 0.7988 | 0.7449 | -0.458 | 0.633 | 0.000726 |
| rs116554979 | 13 | 102123050 | G | 0.9455 | 0.7995 | 1.235 | 3.439 | 0.000166 |
| rs58579040 | 13 | 102128462 | T | 0.9474 | 0.824 | 1.169 | 3.22 | 0.000317 |
| chr13:102131686 | 13 | 102131686 | G | 0.9458 | 0.8037 | 1.097 | 2.996 | 0.000564 |
| rs17623509 | 13 | 102134548 | T | 0.9446 | 0.8203 | 1.012 | 2.752 | 0.000956 |
| rs9513941 | 13 | 102391084 | T | 0.5873 | 0.9755 | 0.375 | 1.456 | 0.000353 |
| rs1304339 | 13 | 102400431 | G | 0.5808 | 0.965 | 0.376 | 1.457 | 0.000358 |
| rs2390650 | 13 | 102400884 | A | 0.5997 | 0.9553 | 0.378 | 1.459 | 0.000397 |
| rs9513942 | 13 | 102402094 | A | 0.592 | 0.9993 | 0.366 | 1.442 | 0.000431 |
| rs9518538 | 13 | 102429665 | G | 0.6101 | 0.9997 | 0.35 | 1.42 | 0.000771 |
| rs9513949 | 13 | 102429844 | A | 0.6101 | 0.9994 | 0.351 | 1.42 | 0.000764 |
| rs10851136 | 13 | 102430263 | G | 0.6106 | 0.9963 | 0.351 | 1.421 | 0.000768 |
| rs1571358 | 13 | 102431036 | G | 0.6117 | 0.994 | 0.354 | 1.424 | 0.000735 |
