# Supplementary material for: Genomewide association study in cervical dystonia demonstrates possible association with sodium leak channel
Source: Mov Disord. 2013 Nov 13;29(2):245–51. doi: 10.1002/mds.25732 (PMC4208301; doi:10.1002/mds.25732)
Supplement: Supplementary file 23 [file mds0029-0245-sd23.docx]

**S-Table 7 Regions not well covered by GWAS and imputation**

| Regions not well covered | | | |
| --- | --- | --- | --- |
| Chr | start | End | region |
| 1 | 121342860 | 142902781 | large peri-centromeric |
| 2 | 90115446 | 95422247 | peri-centromeric |
| 3 | 90425679 | 93520803 | peri-centromeric |
| 4 | 49092676 | 52681128 | peri-centromeric |
| 5 | 46389070 | 49444559 | peri-centromeric |
| 6 | 58773345 | 61946760 | peri-centromeric |
| 7 | 58022575 | 61901780 | peri-centromeric |
| 8 | 43775386 | 46907428 | peri-centromeric |
| 9 | 44899543 | 68160420 | large peri-centromeric |
| 10 | 39058671 | 42638232 | peri-centromeric |
| 11 | 51566909 | 54851226 | peri-centromeric |
| 12 | 34827309 | 37953771 | peri-centromeric |
| 13 | 1 | 19198348 | telomeric plus centromeric |
| 14 | 1 | 20283389 | telomeric plus centromeric |
| 15 | 1 | 20174243 | telomeric plus centromeric |
| 16 | 35198407 | 46539392 | peri-centromeric |
| 17 | 22244412 | 25326941 | peri-centromeric |
| 18 | 15368789 | 18548293 | peri-centromeric |
| 19 | 24490299 | 27771975 | peri-centromeric |
| 20 | 26271277 | 29804073 | peri-centromeric |
| 21 | 1 | 9659762 | telomeric plus centromeric |
| 22 | 1 | 16936098 | telomeric plus centromeric |
