# Supplementary material for: Genomewide association study in cervical dystonia demonstrates possible association with sodium leak channel
Source: Mov Disord. 2013 Nov 13;29(2):245–51. doi: 10.1002/mds.25732 (PMC4208301; doi:10.1002/mds.25732)
Supplement: Supplementary file 24 [file mds0029-0245-sd24.doc]

# SUPPLEMENTARY MATERIALS

### SUPPLEMENTARY METHODS

### Quality control In Genome Studio for cervical dystonia cases

Within Genome Studio, SNPs with call frequency < 0.98 in samples with call rates >95% were re-clustered. After re-clustered, those SNPs with clusters separation < 0.3 and Het excess < -0.1 and > 0.1 were removed. Final samples with call rates <0.975 and SNPs call frequency < 0.99 were excluded.

### Quality control in PLINK for cervical dystonia cases

Samples were checked for overall gender mismatch; relatedness checked by identity-by-descent; and overall sample call rate. One sample from any samples pair with pi-Hat > 0.125 was excluded, removing any relatedness in the range of second to third cousin. Any samples with gender mismatch and call rate < 0.95 were excluded. Population substructure was compared with CEU & TSI population from HapMap3 ^36^. Any samples deviated from the combined CEU TSI sample mean + 6SD were removed (less tight compared with controls as the overall case number was small).

SNPs were checked for Hardy-Weinburg equilibrium (HWE), non-random missingness due to haplotype and phenotype, minor allele frequency (MAF). Any SNPs with HWE p value < 1x10^-6^ , haplotype missingness p value < 1x10^-4^, genotype missingness p value < 1x10^-4^, and MAF < 0.01 were excluded. Phenotype missingness was performed after combined with controls and SNPs with p < 1x10^-4^ were excluded.

### Quality control measure for WTCCC control.

Samples were excluded as suggested by Sanger Institute based on their quality control.

In addition, samples deviated from the combined CEU TSI sample mean + 3SD were removed (Tighter criterion than cases). Any SNPs with, haplotype missingness p value < 1x10^-4^, genotype missingness p value < 1x10^-4^, and MAF < 0.01 were excluded as in cases. SNPs with HWE p value < 1x10^-4^ were excluded (tighter criterion than cases).

### Imputation

1000G 2010 August release ^18^ :

The 2010 August release included 90 Utah residents (CEPH) with Northern and Western European ancestry (CEU), 92 Toscani in Italia (TSI), 43 British in England and Scotland (GBR), 36 Finnish in Finland (FIN), 17 Mexican in Los Angeles, CA (MXL) and 5 Puerto Rican in Puerto Rico (PUR). These made up 283 individuals in the European continental group.

Each chromosome was divided into 10 megabase plus 1 megabase overlapping chunk at both end by splitPed ^37^. Phasing was performed in MaCH v1.0.17 ^38^ with 300 haplotypes considered in each update. 20 iterations were done with no external reference. Other parameters were default. The haplotypes in each chromosome were merged from the 10 megabase chunks in ligateHap ^39^. Overlapping segments were discarded. Imputation on merged haplotypes was done in minimac ^15^ with 10 rounds of optimization of model parameter, taking in 300 haplotypes during parameter optimization using reference taken from the 1000 Genomes Project 2010Aug release.

Association analysis of imputed SNPs was performed in mach2dat, assuming an additive model and regressed phenotype against expected dosage at each imputed SNP with PLINK multi-dimensional scale axis 1 &2 and gender as covariates.

The QC parameter was RSQR as suggested. SNPs with RSQR < 0.3 were excluded and MAF <0.03 were also excluded.

## SUPPLEMENTARY FIGURES

### S-Figure 1.

a) MDS plot of case & control before removal of outlier. b) MDS plot of after removal of cervical dystonia case outlier. c) Enlarged view of MDS plot of London cases and Manchester cases with CEU and TSI. The main bulk of cases from London and Manchester were well mixed and co-localised with CEU. A few London cases were more co-localised with TSI.

### S-Figure 2.

QQ plot for GWAS logistic regression. Lambda was calculated based on median of chi square observed /median of expected using R.

### S-Figure 3.

UK Brain Expression Consortium data--Expression of *NALCN*. *NALCN* is universally expressed in all regions in brain. THAL: thalamus; WHMT: white matter; TCTX: temporal cortex; HIPP: hippocampus; FCTX: frontal cortex; HYPO: hypothalamus; OCTX: occital cortex; MEDU: medulla; SPCO: spinal cord; SNIG: substania nigra; PUTM: putamen; CRBL: cerebellum.

### S-Figure 4.

Locus Zoom Figure of different regions found in table 1 and S table 1. **a) Chr 1 at *RGL1***. **b) Chr 2 at *KIAA1715***. **c) Chr 6 at intergenic at 92Mb. d) Chr 10 intergenic at 28Mb. e) Chr 13 at *COL4A1***. **f) Chr 19 intergenic at 29Mb.**

### S-Figure 5.

Additional Locus Zoom Figure of different regions found in S table 4. **a) Chr 1 at 67Mb. b) Chr 4 at 142Mb. c) Chr 5 at 8Mb. d) Chr 12 at 44 Mb. e) Chr14 at 55 Mb.**
